# Macrophage-specific inhibition of the histone demethylase JMJD3 decreases STING and pathologic inflammation in diabetic wound repair

**DOI:** 10.1038/s41423-022-00919-5

**Published:** 2022-09-20

**Authors:** Christopher O. Audu, William J. Melvin, Amrita D. Joshi, Sonya J. Wolf, Jadie Y. Moon, Frank M. Davis, Emily C. Barrett, Kevin D. Mangum, Hongping Deng, Xianying Xing, Rachel Wasikowski, Lam C. Tsoi, Sriganesh B. Sharma, Tyler M. Bauer, James Shadiow, Matthew A. Corriere, Andrea T. Obi, Steven L. Kunkel, Benjamin Levi, Bethany B. Moore, Johann E. Gudjonsson, Andrew M. Smith, Katherine A. Gallagher

**Affiliations:** 1 Department of Surgery, Section of Vascular Surgery, University of Michigan, Ann Arbor, MI USA; 2 Department of Microbiology and Immunology, University of Michigan, Ann Arbor, MI USA; 3 Department of Medicinal Chemistry, University of Michigan, Ann Arbor, MI USA; 4 Department of Surgery, Section of General Surgery, University of Michigan, Ann Arbor, MI USA; 5 Department of Bioengineering, University of Illinois, Urbana-Champaign, Champaign, IL USA; 6 Department of Dermatology, University of Michigan, Ann Arbor, MI USA; 7 Department of Pathology, University of Michigan, Ann Arbor, MI USA; 8 Department of Surgery, University of Texas Southwestern Medical Center, Dallas, TX USA

**Keywords:** wound healing, epigenetics, diabetes, JMJD3, STING, Chronic inflammation, Epigenetics in immune cells

## Abstract

Macrophage plasticity is critical for normal tissue repair following injury. In pathologic states such as diabetes, macrophage plasticity is impaired, and macrophages remain in a persistent proinflammatory state; however, the reasons for this are unknown. Here, using single-cell RNA sequencing of human diabetic wounds, we identified increased JMJD3 in diabetic wound macrophages, resulting in increased inflammatory gene expression. Mechanistically, we report that in wound healing, JMJD3 directs early macrophage-mediated inflammation via JAK1,3/STAT3 signaling. However, in the diabetic state, we found that IL-6, a cytokine increased in diabetic wound tissue at later time points post-injury, regulates JMJD3 expression in diabetic wound macrophages via the JAK1,3/STAT3 pathway and that this late increase in JMJD3 induces NFκB-mediated inflammatory gene transcription in wound macrophages via an H3K27me3 mechanism. Interestingly, RNA sequencing of wound macrophages isolated from mice with JMJD3-deficient myeloid cells *(Jmjd3*^*f/f*^*Lyz2*^*Cre+*^*)* identified that the STING gene (*Tmem173*) is regulated by JMJD3 in wound macrophages. STING limits inflammatory cytokine production by wound macrophages during healing. However, in diabetic mice, its role changes to limit wound repair and enhance inflammation. This finding is important since STING is associated with chronic inflammation, and we found STING to be elevated in human and murine diabetic wound macrophages at late time points. Finally, we demonstrate that macrophage-specific, nanoparticle inhibition of JMJD3 in diabetic wounds significantly improves diabetic wound repair by decreasing inflammatory cytokines and STING. Taken together, this work highlights the central role of JMJD3 in tissue repair and identifies cell-specific targeting as a viable therapeutic strategy for nonhealing diabetic wounds.

## Introduction

Tissue repair after injury is a highly orchestrated process occurring in overlapping stages of coagulation, inflammation, proliferation, and remodeling [1, 2]. The inflammatory phase is further divided into an early phase, wherein macrophages (Mφs) promote inflammation and pathogen destruction, and a late phase, where Mφs promote tissue repair. We and others have identified that in the setting of type 2 diabetes (T2D), the inflammatory phase is significantly prolonged, which retards progression through the remaining stages of repair. In diabetes, during the inflammatory phase, inflammatory Mvs are initially decreased, resulting in impaired early inflammation, followed by a late, robust Mφ transition to an inflammatory phenotype that persists and does not allow wound healing to progress along its normal course [3–5]. The reasons for this dysregulated inflammation are complex; however, we previously identified epigenetic mechanisms that regulate gene expression in wound Mφs during normal tissue repair and are altered in pathologic states. Despite an increased understanding of the role of epigenetic alterations in wound repair, the precise enzymes involved and their kinetic regulation during normal and diabetic wound repair remain incompletely understood.

We recently identified that the histone demethylase Jumonji domain-containing protein D3 (JMJD3) controls inflammation in vascular disease [6]. Specifically, JMJD3 demethylates the lysine 27 site on histone 3 (H3K27), resulting in the opening of chromatin that renders promoter sites accessible for transcription factor binding [6–8], thus effectively increasing gene expression. Demethylation by JMJD3 at the promoter sites reverses the repressive effect of H3K27 trimethylation (H3K27me3) and results in gene activation and active transcription. We previously found that JMJD3 is increased in the presence of the fatty acid palmitate, which is known to be elevated in obesity-induced diabetes and in diabetic bone marrow progenitor cells [9]. Nevertheless, there remains a dearth of information on JMJD3 in normal and diabetic wound Mφs and its role in inflammation and tissue repair. Given that JMJD3 has been shown to regulate inflammation in other tissues and be increased in diabetic bone marrow, we examined the regulation and role of JMJD3 in wound Mφs during normal and diabetic tissue repair.

Herein, using human wound single-cell RNA sequencing (scRNAseq) and primary wound Mφs isolated from diabetic and control murine wounds, we show that *Jmjd3* in Mφs is necessary for inducing early inflammation in normal wound repair but is pathologically elevated in diabetic Mφs at later stages of repair. Mechanistically, we found that IL-6, a cytokine present at high levels in diabetic peripheral blood and wound tissues, drives the Janus kinase 1,3/signal transducer and activator of transcription 3 (JAK1,3/STAT3) pathway to increase *Jmjd3* expression in diabetic wound Mφs and that abrogation of this pathway leads to decreased Mφ-mediated inflammation in diabetic wounds. Interestingly, wound Mφs from our mice with JMJD3-deficient myeloid cells (*Jmjd3*^*f/f*^*Lyz2*^*Cre+*^) analyzed by bulk RNA-seq showed that JMJD3 regulates the STING gene *(Tmem173)*, which, in addition to NFκB-mediated inflammation, contributes to chronic inflammation in diabetes. Finally, we demonstrated that local, Mφ-specific inhibition of JMJD3 using nanoparticles dramatically improves diabetic wound repair by decreasing NFκB and STING-mediated inflammation. Together, these results suggest a central role for JMJD3 in normal and diabetic wound repair and identify JMJD3 as a viable therapeutic target for nonhealing diabetic wounds.

## Results

### JMJD3 is increased early in wound Mφs, increases NFκB inflammatory cytokine gene expression via H3K27me3 and is regulated by JAK1,3/STAT3 in wound Mφs

**Fig. 1:**
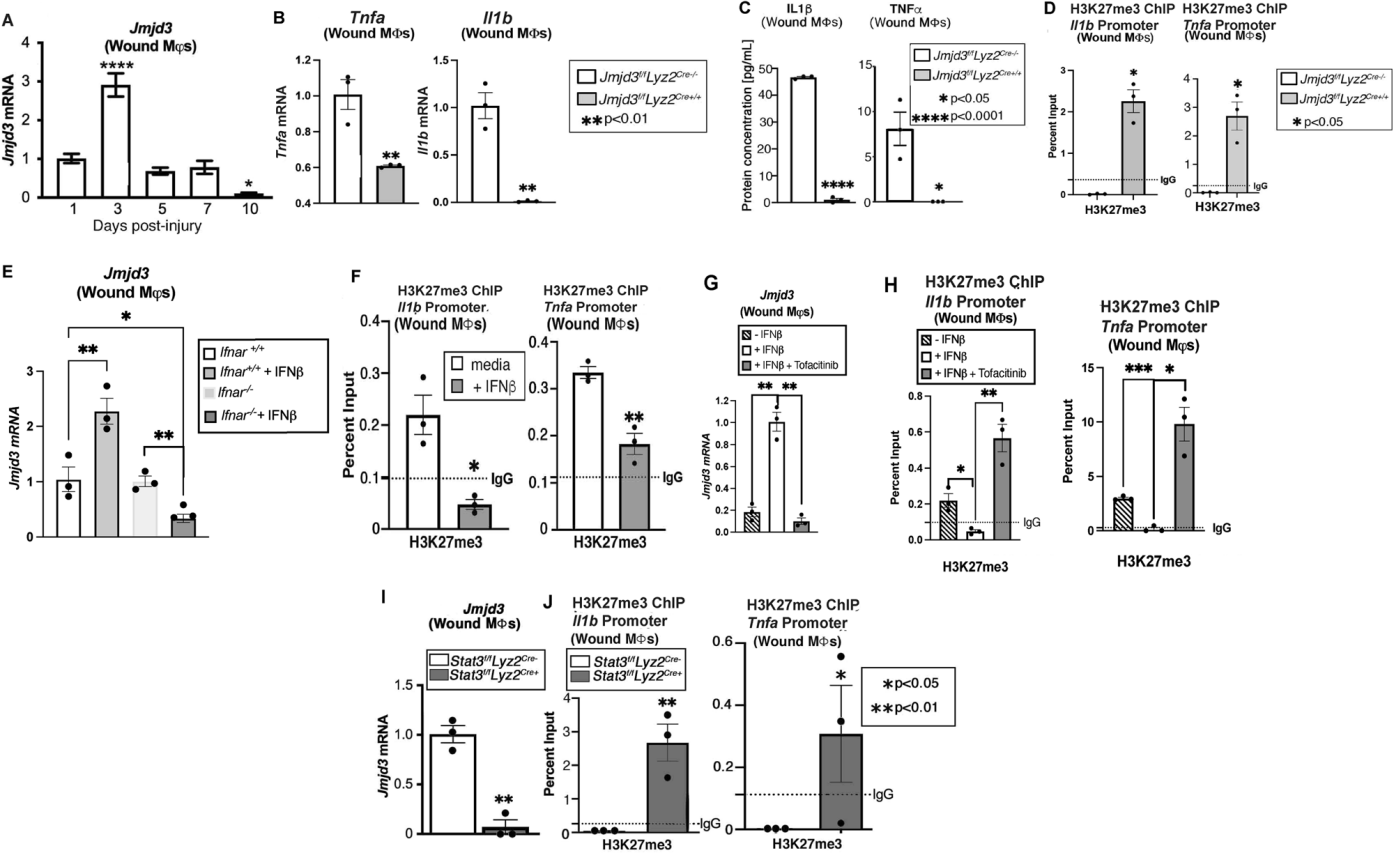
*Jmjd3* directs early inflammation in nondiabetic wound Mφs via the JAK1/3/STAT3 pathway. **A** *Jmjd3* expression from wound Mφs (CD3^-/^CD19^-/^NK1.1^-/^Ly6G^-/^CD11b^+^) harvested on Days 1–10 after wounding (6 mm punch biopsy; N = 4/group, repeated two times). **B** *Tnfa* and *Il1b* expression measured in wound Mφs isolated on Day 3 post-injury from *Jmjd3*^*f/f*^*Lyz2*^*Cre+*^ mice and littermate controls (*N* = 5/group, pooled and repeated in triplicate). **C** Protein levels of TNF-α and IL-1**β** analyzed by ELISAs from *Jmjd3*^*f/f*^*Lyz2*^*Cre+*^ wound Mφs and controls isolated on Day 3 post-injury (*N* = 5/group, pooled and repeated in triplicate). **D** Wound Mφs from *Jmjd3*^*f/f*^*Lyz2*^*Cre+*^ mice and littermate controls were isolated on Day 3, and chromatin immunoprecipitation (ChIP) analysis for H3K7me3 on the *Tnfa* and *Il1b* promoters was performed compared to the IgG control (dotted line). (*N* = 4/group, pooled and repeated in triplicate). **E** Wound Mφs were isolated on Day 3 post-injury from *Ifnar*^−/−^ mice and controls (*Ifnar*^+/+^) and stimulated ex vivo with IFN-**β** (100 U; 8.5 ng/mL) for 6 h, and *Jmjd3* expression was analyzed by RT‒PCR (*N* = 3–5/group, pooled, repeated in triplicate). **F** ChIP analysis of H3K27me3 at the *Il1b* and *Tnfa* promoters from Day 3 isolated wound Mφs following ex vivo IFN-**β** stimulation (100 U; 8.5 ng/mL) for 6 h. **G** *Jmjd3* expression in wound Mφs following ex vivo IFN-**β** stimulation (100 U; 8.5 ng/mL) with and without JAK1,3 inhibition with tofacitinib (100 μM; 6 hr incubation, *N* = 5/group, pooled, repeated in triplicate). **H** ChIP analysis for H3K27me3 at the *Il1b* and *Tnfa* promoters in wound Mφs following ex vivo IFN-**β** stimulation (100 U; 8.5 ng/mL) with and without JAK1,3 inhibition with tofacitinib (100 μM; 6 hr incubation, *N* = 5/group, pooled, repeated in triplicate). **I** *Jmjd3* expression in *Stat3*^*f/f*^*Lyz2*^*Cre+*^ mice and littermate control wound Mφs following ex vivo IFN-**β** stimulation (100 U; 8.5 ng/mL, *N* = 5/group, repeated in triplicate). **H** ChIP analysis of H3K27me3 at the *Il1b* and *Tnfa* promoters in *Stat3*^*f/f*^*Lyz2*^*Cre+*^ wound Mφs following ex vivo IFN-**β** stimulation (100 U; 8.5 ng/mL) for 6 h (*N* = 5/group, pooled, repeated in triplicate). *p < 0.05, **p < 0.01, ***p < 0.001, ****p < 0.0001. Data are presented as the mean ± SEM. Data were first analyzed for a normal distribution, and if the data passed the normality test, a two-tailed Student’s *t* test was used

Epigenetic-based histone modifications have been shown by our group and others to regulate Mφs phenotype and function during wound repair [10–12]. JMJD3 plays a vital role in inflammation in other vascular tissues, and given the importance of Mφ-mediated inflammation in wound repair, we investigated JMJD3 in wound Mφs following injury. First, to establish the normal kinetics of JMJD3 following injury, we sorted wound Mφs (CD3^-^/CD19^-^/NK1.1^-^/Ly6G^-^/CD11b^+^) daily for 10 days following wounding (6 mm punch biopsy) and analyzed *Jmjd3* expression. We identified a significant increase in *Jmjd3* on Day 3 post-injury, followed by a rapid decrease over the course of repair (Fig. 1A). By Day 5 (following the acute inflammatory phase), *Jmjd3* expression in wound Mφs had returned to baseline levels. Next, to examine the relevance of JMJD3 in wound Mφs during repair, we generated a myeloid-specific, JMJD3-deficient mouse strain using the Cre recombinase Lox-P system [13] *(Jmjd3*^*f/f*^*Lyz2*^*Cre+*^*)*. Confirmation of reduced *Jmjd3* specifically in wound Mφs isolated from the *Jmjd3*^*f/f*^*Lyz2*^*Cre+*^ mice was obtained (Supplementary Fig. 1A).

Given that we and others have shown that NFκB-mediated inflammation is important in wound Mφs and is often epigenetically regulated, we specifically examined the expression of NFκB-mediated genes previously identified to be important for wound repair (e.g., *Il1b, Tnfa*) in isolated wound Mφs from the *Jmjd3*^*f/f*^*Lyz2*^*Cre+*^ mice and littermate controls at Day 3 post-wounding. As expected, we identified significant decreases in *Il1b* and *Tnfa* as well as *Il12 and Il23 e*xpression in the wound Mφs from the *Jmjd3*^*f/f*^*Lyz2*^*Cre+*^ mice (Fig. 1B, Supplementary Fig. 1B). This phenomenon reduced *Il1b and Tnfa* expression in the *Jmjd3*^*f/f*^*Lyz2*^*Cre+*^ wound Mφs and was also observed at the protein level by ELISAs (Fig. 1C). Since JMJD3 alters gene expression through demethylation of H3K27me3, we examined wound Mφs from the *Jmjd3*^*f/f*^*Lyz2*^*Cre+*^ mice by chromatin immunoprecipitation (ChIP) for H3K27me3 at the NFκB binding sites of the *Il1b and Tnfa* gene promoters. We found a significant increase in the repressive H3K27me3 mark at these NFκB binding sites on the promoters of *Il1b and Tnfa* (Fig. 1D, Supplemental Fig. 1C).

Our group previously showed that Type 1 interferons (IFN-I), such as IFN-β, are increased in normal wound tissue early during the inflammatory phase of wound repair [4, 11]. Although IFN-β can activate numerous downstream pathways, one of the more commonly studied pathways involves the activation of Janus kinase (JAK) proteins, which leads to tyrosine phosphorylation that, in turn, activates signal transducers and activators of transcription (STAT) proteins and translocates dimerized, phosphorylated proteins to the nucleus [14–16]. Furthermore, IFN-β has been shown by our group and others to modulate epigenetic enzyme expression in wounds and other vascular tissues, where they function as transcriptional regulators [3, 12, 17]. Given the known early spike of IFN-β in wound tissue undergoing normal repair processes, we examined the effect of IFN-β stimulation on *Jmjd3* in wound Mφs. Wound Mφs were sorted on Day 3 post-injury, stimulated ex vivo with IFN-β (100 U/mL; 8.5 ng/mL) for 6 h and analyzed for *Jmjd3* expression. *Jmjd3* expression was significantly increased in wound Mφs following IFN-β stimulation, and this effect was abrogated in wound Mφs sorted from the mice deficient in the IFN-αβ receptor (*Ifnar*^−/−^) (Fig. 1E). To examine the downstream effects of IFN-β stimulation on JMJD3 and H3K27me3 regulation of inflammatory cytokines, we isolated wound Mφs ex vivo, treated them with IFN-β and examined them by ChIP analysis for H3K27me3 at NFκB binding sites on promoters of inflammatory genes important for wound repair (e.g., *Il1b, Tnfa*). We found that levels of H3K27me3 were significantly decreased at NFκB binding sites on *Il1b* and *Tnfa* gene promoters following IFN-β stimulation of wound Mφs (Fig. 1F). Next, to determine whether IFN-β regulates *Jmjd3* expression via JAK/STAT signaling, we examined JAK1, JAK3 and STAT3 levels following IFN-β stimulation of wound Mφs ex vivo. We confirmed prior reports [18, 19] that IFN-β increases JAK1 and 3 and STAT3 phosphorylated protein levels, and this effect is negated in *Ifnar*^−/−^ mice (Supplementary Fig. 2). To study the effect of JAK1/3 on *Jmjd3* expression in wound Mφs, we used tofacitinib, a commercially available competitive inhibitor of JAK1/3, and treated isolated wound Mφs ex vivo with IFN-β and tofacitinib (100 μM) for 6 h. We found that inhibition of JAK1/3 led to significantly decreased levels of *Jmjd3* in the wound Mφs (Fig. 1G), and when we examined the *Il1b* and *Tnfa* gene promoters by ChIP, we found that JAK1/3 blockade resulted in increased H3K27me3 at the NFκB binding sites of these genes, resulting in decreased gene activation (Fig. 1H). Next, we studied the effects of STAT3 on *Jmjd3* expression in wound Mφs. We used the Cre/LoxP system to generate myeloid-depleted STAT3 mice (*Stat3*^*f/f*^*Lyz2*^*Cre+*^) as previously described [13]. Wound Mφs (CD3^-^/CD19^-^/NK1.1^-^/Ly6G^-^/CD11b^+^) isolated on Day 3 from myeloid-cell STAT3-deficient mice were isolated by sorting and stimulated ex vivo with IFN-β for 6 h. These Mφs exhibited significantly decreased *Jmjd3* expression (Fig. 1I), and ChIP analysis of NFκB-mediated inflammatory promoters showed that *Il1b* and *Tnfa* had increased H3K27me3 levels (Fig. 1J). Taken together, these data suggest that the increased IFN-β observed in early wound inflammation regulates early JMJD3 expression in normal wound Mφs via the JAK1/STAT3/STAT3 pathway.

### JMJD3 is increased in diabetic wound Mφs during the late inflammatory phase

**Fig. 2:**
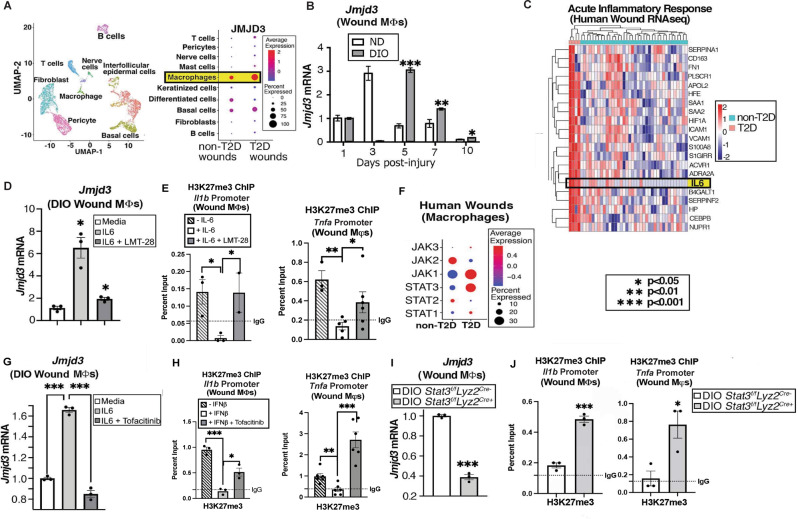
*Jmjd3* increases late in diabetic wound Mφs. **A** Cluster analysis UMAP of single-cell RNA sequencing from human T2D and non-T2D wounds showed 10 unique cell clusters (representative). Dot plot demonstrating JMJD3 expression within the Mφ population in human T2D and non-T2D wound samples (*N* = 42). The dot size corresponds to the proportion of cells within the group expressing each transcript, while the dot color corresponds to the expression level. **B** DIO and ND wound Mφs (CD3^-^/CD19^-^/NK1.1^-^/Ly6G^-^/CD11b^+^) were harvested on Days 1–10 and analyzed for *Jmjd3* expression by RT‒PCR (*N* = 4/group, repeated once). **C** Human bulk RNA sequencing heatmap reflecting the expression profiles for selective genes (rows) across different samples (columns; stratified by T2D status) from acute inflammatory response Gene Ontology pathway analysis with upregulation of IL-6 in T2D wounds compared to control wounds (*N* = 42). **D** DIO wound Mφs were isolated on Day 5, treated ex vivo for 6 h with recombinant IL-6 (rIL-6; 20 nM) with and without IL-6 receptor inhibition (LMT-28; 200 nM) and analyzed for *Jmjd3* expression (*N* = 3–5/group, pooled, repeated in triplicate). **E** ChIP analysis for H3K27me3 on the *Tnfa* and *Il1b* promoters from diabetic wound Mφs with and without IL-6 receptor inhibition (LMT-28; 200 nM; *N* = 3–5/group, pooled and repeated in triplicate). **F** Human single-cell RNA sequencing dot plot demonstrating *Jak*/*Stat* gene expression within the Mφ population in human T2D and non-T2D wound samples. (Cluster analysis UMAP shown above in (**A**)). The dot size corresponds to the proportion of cells within the group expressing each transcript, while the dot color corresponds to the expression level. **G** *Jmjd3* expression following rIL-6 stimulation (20 nM) and Jak1/3 inhibition (tofacitinib; 100 μM) in diabetic wound Mφs isolated from Day 5 wounds and treated ex vivo for 4 h. (*N* = 5/group, pooled and repeated in triplicate). **H** ChIP analysis for H3K27me3 on the *Tnfa* and *Il1b* promoters from diabetic wound Mφs following ex vivo rIL-6 stimulation (20 nM) with and without JAK1/3 inhibition (tofacitinib; 100 μM) for 4 h (*N* = 5/group, pooled, repeated in triplicate). **I** *Jmjd3* expression in DIO *Stat3*^*f/f*^*Lyz2*^*Cre+*^ and DIO littermate control wound Mφs following ex vivo rIL-6 stimulation (20 nM) for 6 h. (*N* = 3/group, pooled, repeated in triplicate). **J** ChIP analysis for H3K27me3 on *Tnfa* and *Il1b* promoters from DIO *Stat3*^*f/f*^*Lyz2*^*Cre+*^ and DIO littermate control wound Mφs following rIL-6 stimulation (20 nM) for 6 h (*N* = 4/group, pooled, repeated in triplicate). Data are presented as the mean ± SEM. All data are representative of 2–4 independent experiments

Given that NFκB-mediated inflammatory cytokines have been shown to be elevated in diabetic wounds [3, 5, 20] and that JMJD3 regulates inflammatory cytokines in normal wound Mφs, we examined whether JMJD3 was elevated in diabetic wound Mφs. We determined the translational relevance by first examining human wound Mφs analyzed previously by our group [10] using single-cell RNA sequencing (scRNA-seq) from T2D and non-T2D patients and found that JMJD3, along with IL-6, TNF-α, IL-1β, and IL23, was significantly increased in Mφs from chronic wounds of T2D patients (Fig. 2A, Supplementary Fig. 3). Similarly, we generated a murine model of obesity and T2D by administering a high-fat diet chow (60% carbohydrates versus 12% in normal chow) for 12-20 weeks. These mice with diet-induced obesity (DIO) exhibit insulin resistance and impaired glucose tolerance [21]. Wound Mφs (CD3^-^/CD19^-^/NK1.1^-^/Ly6G^-^/CD11b^+^) were isolated every 48 h post-wounding from the mice with DIO and demonstrated increased *Jmjd3* expression on Days 5–10 during the late stages of wound repair, when wound Mφs should have transitioned from a pro- to an anti-inflammatory phenotype (Fig. 2B). Furthermore, we noted a sustained elevation of the inflammatory genes *Il6*, *Il1b*, and *Tnfa* in diabetic wound Mφs, while *Ifnb* was significantly reduced on Day 1 in diabetic wound Mφs and remained unchanged or decreased relative to that of nondiabetic wounds throughout the time course (Supplementary Fig. 4A–D), suggesting that early IFN-β production in wounds suppresses inflammatory cytokine production and is necessary for healthy wound repair.

Since we and others have previously shown that diabetic wounds fail to upregulate IFN-β post-injury and during wound repair [11, 22–24], we examined our previously published human wound bulk RNA-seq dataset for other potential cytokines/chemokines that are elevated in diabetic wound tissue and may regulate the JAK/STAT pathway and JMJD3 in diabetic wound Mφs [10]. As others have previously identified [25, 26], we found increased IL-6 in human T2D wounds (Fig. 2C). Mechanistically, IL-6 binds to the IL-6 receptor (IL-6R), which is common on Mφ cell surfaces, and this complex then couples with the transmembrane spanning IL-6R subunit-b gp-130. Binding with gp-130 facilitates homodimerization that leads to stimulation of the JAK/STAT3 pathway downstream [18, 27]. Importantly, IL-6 has been shown to specifically upregulate STAT3 compared to other STAT proteins [28–30]. To directly examine the effects of IL-6 on JMJD3 in diabetic wound Mφs, we isolated wound Mφs from the mice with DIO and treated them ex vivo with recombinant IL-6 (rIL-6; 20 nM). We observed a significant increase in *Jmjd3* expression in DIO wound Mφs after treatment with rIL-6, and this effect was abrogated with an IL-6 receptor inhibitor (200 nM), LMT-28, a small molecule that competitively blocks phosphorylation of the IL-6 receptor beta subunit glycoprotein-130 [31] (Fig. 2D). To identify whether IL-6 could upregulate inflammatory genes via a JMJD3/H3K27me3 mechanism, we examined DIO wound Mφs isolated and treated them ex vivo with rIL-6, with or without LMT-28. We observed increased H3K27me3 repressive marks at inflammatory cytokine promoters (e.g., *Il1b, Tnfa*) with IL-6R inhibition using LMT-28 (Fig. 2E).

We then examined whether downstream IL-6 receptor signaling activated JAK/STAT3 to increase *Jmjd3* in diabetic wounds. First, we analyzed our human wound scRNA-seq data [10] and found that JAK1 and STAT3 were strongly elevated in T2D wound Mφs compared to non-T2D wounds (Fig. 2F). DIO wound Mφs were treated ex vivo with rIL-6 and the JAK1/3 inhibitor tofacitinib (100 μM); *Jmjd3* expression increased with rIL-6 treatment of DIO wound Mφs, and this effect was reversed after treatment with tofacitinib in the presence of rIL-6 (Fig. 2G). A concomitant increase in H3K27me3 at the *Il1b* and *Tnfa* promoters when examined by ChIP (Fig. 2H) was noted with tofacitinib treatment. We also examined the role of STAT3 by isolating wound Mφs from *Stat3*^*f/f*^*Lyz2*^*Cre+*^ mice on a high-fat diet for 12-20 weeks (DIO *Stat3*^*f/f*^*Lyz2*^*Cre+*^) on Day 5 post-injury and treating them ex vivo with rIL-6. In these cells, we observed a decrease in *Jmjd3* expression (Fig. 2I) and an increase in H3K27me3 at the *Il1b* and *Tnfa* promoter binding sites when examined by ChIP (Fig. 2J). Together, these data suggest that in diabetic wound tissue, JMJD3 increases in the later stages of wound healing, is regulated by IL-6 through a JAK1/3/STAT3 pathway, and, as such, may be partially responsible for the persistent inflammatory phenotype seen in diabetic wound Mφs.

### JMJD3 regulates STING in pathologic wound Mφs

**Fig. 3:**
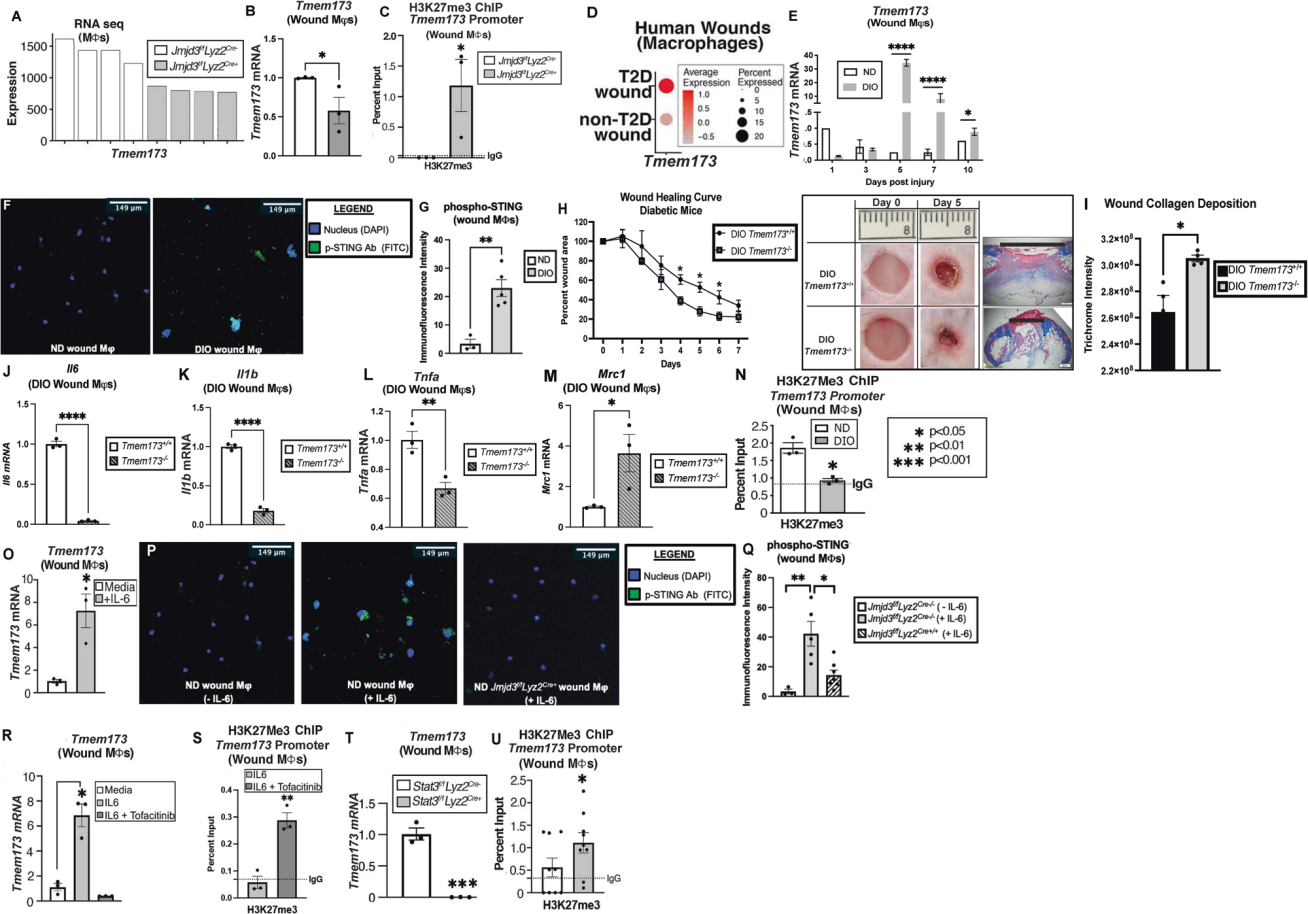
STING-mediated inflammation is regulated by JMDJ3 in wound Mφs. **A** Bulk RNA sequencing analysis of wound Mφs (CD3^-/^CD19^-/^NK1.1^-/^Ly6G^-/^CD11b^+^) isolated on Day 5 from *Jmjd3*^*f/f*^*Lyz2*^*Cre+*^ mice and littermate controls with *Tmem173* gene expression (*N* = 4/group). **B**, **C** *Tmem173* expression and ChIP analysis of H3K27me3 at the *Tmem173* promoter in *Jmjd3*^*f/f*^*Lyz2*^*Cre+*^ and littermate control wound Mφs (*N* = 4/group, pooled, repeated in triplicate). **D** Human single-cell RNA sequencing dot plot demonstrating *Tmem173* gene expression within the Mφ population in human T2D and non-T2D wound samples. Cluster analysis UMAP shown above in (**2** **A**). The dot size corresponds to the proportion of cells within the group expressing each transcript, while the dot color corresponds to the expression level. **E** *Tmem173* gene expression from murine diabetic wound Mφs harvested on Days 1–10 compared with that of the nondiabetic controls (*N* = 4/group, repeated once). **F** Immunofluorescence against phospho-STING antibody (FITC) in ND and DIO wound Mφs. **G** Quantification of the immunofluorescence intensity of phospho-STING (*N* = 3–5 cells/group). **H** Wound healing curve for DIO STING knockout (DIO *Tmem173*^−/−^*)* mice and littermate controls (DIO *Tmem173*^*+/+*^*)*, with representative healing images on Days 0 and 5 post-injury (6 mm punch wounds; 3–4 mice/group; repeated once). Wounds were harvested on Day 5, paraffin embedded, and stained with Masson’s trichrome stain (*N* = 3 mice/group). Representative images are shown at ×2 magnification. The black horizontal bar above the wound represents the entire wound distance, the epithelial tongues are denoted by arrowheads, and the asterisk (*) denotes wound debris. The scale bar represents 500 μm. **I** Collagen quantification of trichrome staining in DIO *Tmem173*^+/+^ and DIO *Tmem173*^−/−^ mice (*N* = 4 wounds/strain; repeated once). **J** *Il6* mRNA expression in Day 5 wound Mφs from DIO *Tmem173*^+/+^ and DIO *Tmem173*^-/-^ mice. (*N* = 4 mice/group, pooled, repeated in triplicate). **K** *Il1b* mRNA expression in Day 5 wound Mφs from DIO *Tmem173*^+/+^ and DIO *Tmem173*^−/−^ mice. (*N* = 4 mice/group, pooled, repeated in triplicate). **L** *Tnfa* mRNA expression in Day 5 wound Mφs from DIO STING^+/+^ and DIO STING^−/−^ mice^.^ (*N* = 4 mice/group, pooled, repeated in triplicate). **M** *Mrc1* mRNA expression in Day 5 wound Mφs from DIO *Tmem173*^+/+^ and DIO *Tmem173*^−/−^ mice. (*N* = 4 mice/group, pooled, repeated in triplicate). **N** ChIP analysis of H3K27me3 at the *Tmem173* gene promoter in ND and DIO wound Mφs (*N* = 4/group, pooled, repeated in triplicate). **O** *Tmem173* expression in wound Mφs following rIL-6 stimulation (20 nM) for 4 h (*N* = 5/group, pooled, repeated in triplicate). **P** Immunofluorescence against phospho-STING antibody (FITC) in ND wound Mφs ± rIL-6 stimulation (20 nM; 1 hr) and in *Jmjd3*^*f/f*^*Lyz2*^*Cre+*^ wound Mφs treated with rIL-6 (20 nM; 1 h). **Q** Quantification of the immunofluorescence intensity of phospho-STING (FITC) by ImageJ (NIH) (*N* = 3–5 cells/group). **R** *Tmem173* expression in wound Mφs following rIL-6 stimulation (20 nM) for 6 h with and without JAK1/3 inhibition (tofacitinib; 100 μM; *N* = 3/group, pooled, repeated in triplicate). **S** ChIP analysis of H3K27me3 on the *Tmem173* promoter in wound Mφs following rIL-6 stimulation (20 nM) for 4 h with and without Jak1/3 inhibition (tofacitinib; 100 **μ**M, *N* = 4/group, pooled, repeated in triplicate). **T** *Tmem173* expression from *Stat3*^*f/f*^*Lyz2*^*Cre+*^ and littermate control wound Mφ following rIL-6 stimulation (20 nM) for 4 h (*N* = 3/group, pooled, repeated in triplicate). **U** ChIP analysis of H3K27me3 on the *Tmem173* gene promoter from *Stat3*^*f/f*^*Lyz2*^*Cre+*^ and littermate control wound Mφs following rIL-6 stimulation (20 nM) for 4 h (*N* = 6/group, pooled, repeated in triplicate). *p < 0.05, **p < 0.01, ***p < 0.001. Data are presented as the mean ± SEM

To identify additional relevant genes that *Jmjd3* may regulate in wound repair, we isolated wound Mφs (CD3^-^/CD19^-^/NK1.1^-^/Ly6G^-^/CD11b^+^) from myeloid-depleted JMJD3 mice (*Jmjd3*^*f/f*^*Lyz2*^*Cre+*^) and littermate controls and performed bulk RNA sequencing analysis. We then examined this dataset for differentially regulated genes that were previously associated with inflammation and/or wound repair. This analysis revealed that in JMJD3-deficient mice, in addition to other inflammatory genes, *Tmem173*, a gene involved in the cytosolic GMP-AMP synthase - stimulator of interferon genes (cGAS-STING) inflammatory pathway, was significantly decreased (Fig. 3A). cGAS-STING has been shown to sustain chronic inflammation in pathologic conditions such as obesity-induced diabetes [32–35], myocardial infarction [17], and chronic inflammatory diseases [36–39]. Although the downstream effects of STING-mediated inflammation in cancer and other chronic inflammatory conditions have been extensively studied, the upstream regulation of *Tmem173* gene expression is unknown.

To further investigate our findings from the RNA-seq data, we isolated wound Mφs from our *Jmjd3*^*f/f*^*Lyz2*^*Cre+*^ mice and littermate controls and found decreased *Tmem173* in the JMJD3-deficient wound Mφs, consistent with our RNA-seq data (Fig. 3B). Furthermore, ChIP analysis at the GATA1 binding site on the *Tmem173* gene promoter revealed a concomitant increase in the H3K27me3 mark at the *Tmem173* promoter in wound Mφs (Fig. 3C). Additionally, when we examined scRNA-seq data from human T2D and non-T2D wounds [10], we observed elevated *Tmem173* in the human T2D wound Mφs (Fig. 3D). We studied the kinetics of *Tmem173* expression in diabetes by isolating wound Mφs from the mice with DIO and their ND controls up to 10 days after wounding and analyzed them for *Tmem173* expression. We observed that in DIO wound Mφs, *Tmem173* increased late in the wound healing process (Fig. 3E) compared to that of the littermate controls. Notably, the slight increase observed in *Tmem173* at Day 10 in the ND wound Mφs did not translate to the protein level (Supplementary Fig. 5A). Wound Mφs obtained on Day 5 post-wounding revealed that the mice with DIO had significantly increased phosphorylated STING protein levels (p-STING), as shown by immunofluorescence (Fig. 3F, G), and STING levels, as shown by Western blots (SupplementARY Fig. 5B).

Diabetic STING knockout mice, *Tmem173*^*–/–*^ (DIO STING^−/−^), were generated by subjecting *Tmem173*^–/–^ mice to a high-fat diet for 12-20 weeks, and a wound healing curve was obtained against age-matched littermate controls. The *Tmem173*^−/−^ mice with DIO exhibited improved wound healing at Days 4–7 (Fig. 3H), correlating with the days of greatest *Tmem173* expression. Histologic examination of Day 5 wounds from the DIO STING^-/-^ mice and the control mice revealed higher collagen deposition by trichrome staining and improved wound contraction (Fig. 3I). Next, we examined the role of STING in promoting downstream inflammation in wound Mφs and noted that in DIO *Tmem173*^−/−^ wound Mφs isolated on Day 5 post-injury, there was a significant decrease in the inflammatory cytokines *Il6, Il1b, and Tnfa*, with a concomitant increase in the reparative gene mannose receptor complex 1 (*Mrc1)* (Fig. 3J–M). Examination of nondiabetic wound Mφs revealed that in the *Tmem173*^−/−^ mice, there was a significant increase in the inflammatory cytokines *Il1b, Il6*, and *Tnfa* (Supplementary Fig. 6A–C) with nonsignificant changes in *Mrc1*, suggesting a context-specific, dichotomous role for STING in regulating Mφ-mediated inflammatory cytokines and wound repair (Supplementary Fig. 6D, E).

To explore this issue further, we examined downstream STING pathway signaling by western blotting for the levels of p-IRF3, p-TBK1 and p-NFκB (p65) in wound Mφs from the nondiabetic *Tmem173*^−/−^ mice and their littermate controls. We noted that wound Mφs from the *Tmem173*^−/−^ mice displayed decreased p-IRF3, p-TBK1 and p-NFκB levels (Supplementary Fig. 7A–D), suggesting that in the nondiabetic state, STING may regulate the early production of IFN-I and signals primarily through a TBK1/IRF3 axis, contributing to normal wound repair. As expected, a wound healing curve of the *Tmem173*^−/−^ mice showed impaired wound healing compared to that of the littermate controls, further suggesting that a baseline level of STING activity is necessary for normal wound repair (Supplementary Fig. 6E).

We then examined the downstream STING pathway in the setting of diabetes. In addition to the decreased inflammatory markers noted in DIO *Tmem173*^−/−^ wound Mφs (Fig. 3J–M), Western blotting revealed that DIO *Tmem173*^*-/-*^ wound Mφs, at baseline and with rIL-6 stimulation, exhibited increased p-IRF3, p-TBK1 and p-NFκB expression (Supplementary Fig. 7E–H) compared to the controls. These data suggest that in the diabetic state, STING may have reduced effects on the TBK1/IRF3/IFN-I pathway.

Subsequently, we explored the role of JMJD3 in the upstream regulation of *Tmem173* expression in diabetic wound Mφs. We observed significantly decreased levels of the transcriptionally repressive H3K27me3 mark at the *Tmem173* promoter in diabetic wound Mφs compared to the normal diet controls (Fig. 3N). Examination of other histone methylation and acetylation marks at the *Tmem173* promoter did not show other transcriptionally activating modifications (Supplementary Fig. 5C, D). To simulate diabetic wound inflammatory cytokine expression conditions in our mouse model, we stimulated Day 5 wound Mφs from normal diet-fed mice ex vivo with rIL-6 (20 nM), and increased *Tmem173* expression was observed (Fig. 3O). When these Mφs were examined by immunofluorescence, there was significantly increased phospho-STING staining that was abrogated in the *Jmjd3*^*f/f*^*Lyz2*^*Cre+*^ wound Mφs (Fig. 3P, Q). We then investigated whether upstream blockade of JMJD3 production affects *Tmem173* transcription. Wound Mφs were isolated at 5 days post-wounding and stimulated with rIL-6 agent for 6 h with and without the JAK1/3 inhibitor tofacitinib (100 μM). Quantitative PCR analysis revealed decreased *Tmem173* expression (Fig. 3R) and a concomitant increase in the H3K27me3 mark at the *Tmem173* promoter binding site when wound Mφs were treated with rIL-6 and tofacitinib (Fig. 3S). Furthermore, when wound Mφs from myeloid-depleted STAT3 mice *(Stat3*^*f/f*^*Lyz2*^*Cre+*^*)* were isolated and treated ex vivo with rIL-6, they exhibited decreased *Tmem173* expression (Fig. 3T) and increased H3K27me3 marks at the *Tmem173* promoter binding site (Fig. 3U) compared to those of their littermate controls. Finally, we observed that myeloid depletion of *Jmjd3* in diabetes results in increased production of the anti-inflammatory cytokine IL10 (Supplementary Fig. 8A), which results in elevated levels of reparative, anti-inflammatory Ly6C^Lo^ CD11b^+^ Mφs critical for tissue repair (Supplementary Fig. 8B, analyzed by flow cytometry as previously described by our laboratory [4]). Taken together, these results suggest that JMJD3 regulates STING expression and thereby influences the Mφ-mediated inflammatory phenotype in wound healing.

### Macrophage-specific JMJD3 inhibition improves tissue repair in diabetic wounds

**Fig. 4:**
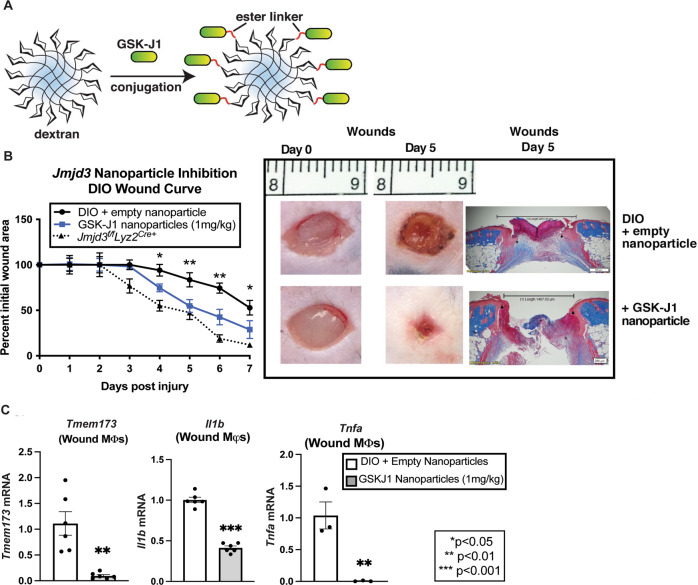
Mφ-specific nanotherapy against JMJD3 improves diabetic tissue repair. **A** Schematic of Mφ specific, GSK-J1-laden, dextran core nanoparticles. **B** Wound healing curve for DIO mice wounded with a 6 mm punch biopsy, and wounds were injected daily starting on Day 1 post-injury with nanoparticles containing either a selective JMJD3 inhibitor (GSK-J1; 1 mg/kg) or dextrose control. DIO mice deficient in myeloid JMJD3 production *(Jmjd3*^*f/f*^*Lyz2*^*Cre+*^*)* were included as a control. Wound area was measured daily with ImageJ software throughout the wound healing course (*N* = 5/group, repeated twice). Representative wound images at ×2 magnification on Day 0 and Day 5 are shown. Wounds were harvested on Day 5, paraffin embedded, and stained with Masson’s trichrome stain (*N* = 5–6 mice/group). The black bar above the wound represents the entire wound distance, the arrowheads represent the epithelial tongue edges, and the asterisk (*) represents wound debris. The scale bar represents 200 μm. **C** *Tmem173, Il1b and Tnfa* expression in DIO wound Mφs (CD3^-^/CD19^-^/NK1.1^-^/Ly6G^-^/CD11b^+^) harvested on Day 5 from wounds treated with and without GSK-J1 nanoparticles (*N* = 3–4/group, pooled, repeated in triplicate). *p < 0.05, **p < 0.01. Data are presented as the mean ± SEM. Data were first analyzed for normal distribution, and if data passed the normality test, a two-tailed Student’s *t* test was used

Given the central role of JMJD3 activity in Mφ-mediated inflammation in tissue repair and its pathologic role in sustaining inflammation in diabetic wounds, we theorized that cell-specific targeting of JMJD3 would be a viable therapeutic target. To examine this, we synthesized Mφ-targeting nanoparticles [10, 40] by linking dextran-laden nanoparticles with a known JMJD3 covalent inhibitor, GSK-J1 [41] (Fig. 4A). Dextran is known to be efficiently taken up by tissue Mφs due to binding of the mannose receptor (CD206) and is used as a standard label for Mφs in animal models [40, 42–44]. First, we verified their Mφ specificity (Supplementary Fig. 9) and then performed subcutaneous injections (1 mg/kg) into the wounds of the mice with DIO daily following wounding for 7 days. Wound healing, compared to that of the placebo-treated mice and the *DIO Jmjd3*^*f/f*^*Lyz2*^*Cre+*^ mice (phenotypic control), was monitored and analyzed by ImageJ software and histology. We observed that Mφ-specific nanoparticle inhibition of JMJD3 resulted in significantly improved wound healing in the GSK-J1 nanoparticle-treated group and was most pronounced in the later days of wound healing. This period is consistent with the days of highest *Jmjd3* and *Tmem173* expression in diabetic wound Mφs. For comparison, the *DIO Jmjd3*^*f/f*^*Lyz2*^*Cre+*^ mice exhibited improved wound healing similar to the nanoparticle-treated cohort. Histologically, wounds harvested on Day 5 of nanoparticle treatment had increased epithelialization compared to the placebo controls, signifying advancement through the normal stages of tissue repair in the treated wounds (Fig. 4B). To confirm that the observed effects were mediated by *Jmjd3* inhibition in Mφs, we isolated wound Mφs (CD3^-^/CD19^-^/NK1.1^-^/Ly6G^-^/CD11b^+^) from the nanoparticle-treated mice and their controls on Day 5 after wounding and analyzed them. These assays revealed significantly decreased *Tmem173, Il1b* and *Tnfa* expression in the wound Mφs from treated wounds compared to the placebo-treated wound Mφs by gene expression and protein levels (Fig. 4C; Supplementary Fig. 10). Taken together, these data suggest that Mφ-specific targeting of JMJD3 in diabetic wounds ameliorates persistent inflammation resulting from the NFκB and STING pathways and results in improved diabetic wound repair, making this a viable therapeutic strategy.

## Discussion

**Fig. 5:**
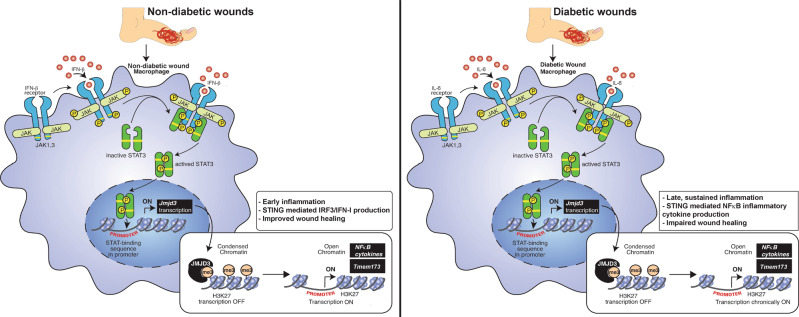
Schematic of the mechanism of JMJD3 activity in wound Mφs. (Left) Nondiabetic wound Mφs are stimulated by IFN-β, which acts through the JAK/STAT3 pathway to stimulate the early transcription of JMJD3 and facilitate early inflammation. This cascade primes STING toward a TBK1/IRF3/IFN-I pathway and ultimately leads to wound repair. (Right) In diabetic wounds, Mφs are stimulated by IL-6 through a JAK/STAT3 pathway to stimulate the late transcription of JMJD3, leading to late, sustained inflammation. This cascade primes STING toward diminished IFN-I production and an increased NFκB/inflammatory cytokine pathway that leads to an inflammatory Mφ phenotype and impaired diabetic wound healing

Nonhealing diabetic wounds are highly prevalent, with limited therapeutic options [45, 46]. Here, we showed that JMJD3 is critical for regulating Mφ-mediated inflammation in normal wound repair. We also identified that persistent, pathologic inflammation, as seen in T2D, is mediated by the expression of JMJD3 in Mφs past the initial inflammatory phase of wound repair. Upregulation of JMJD3 in diabetic wound Mφs led to decreased repressive H3K27 trimethylation at NFκB binding sites at the promoters of inflammatory genes, resulting in increased inflammatory gene transcription. Herein, using human wound Mφs from chronic diabetic wounds and wound Mφs from DIO murine models, we show that JMJD3 is regulated by the JAK1,3/STAT3 pathway, whereas in diabetic wounds, IL-6 is the primary driver of JAK1,3/STAT3 signaling in Mφs (Fig. 5). Furthermore, RNA sequencing analysis of Mφs from myeloid cell-deficient JMJD3 mice showed that JMJD3 regulates *Tmem173*, which is known to perpetuate chronic inflammation in T2D [32, 36, 37]. We found that *Tmem173* is elevated in diabetic wound Mφs and contributes to impaired wound healing by decreasing TBK1/IRF3/IFN-I signaling. Finally, we found that Mφ-specific nanoparticle inhibition of JMJD3 in diabetic wounds led to decreased inflammatory cytokine production and improved tissue repair. Together, this work highlights the central role of JMJD3 in normal and pathologic wound healing.

Initial investigations into the role of JMJD3 in wound healing have revealed that global JMJD3 inhibition leads to impaired keratinocyte migration and decreased epithelial maturation, resulting in poor wound healing [47–49]. These studies examined JMJD3 in keratinocyte functions and identified that JMJD3 played a role in wound repair via Notch activation in keratinocytes. A more recent report examined the role of microRNA regulation of JMJD3 in human keratinocytes and fibroblasts during the early phase of wound healing [48]. This study showed that JMJD3 is necessary for keratinocyte function in early wound healing, which correlates with our findings on the importance of JMJD3 in Mφs in the acute inflammatory phase of wound healing. Finally, a number of studies have examined the role of JMJD3 in regulating fibrosis, a key component of the tissue repair cascade, following myocardial infarction [50], in hepatocytes [51] and in diabetic renal tissue [52]. In these studies, JMJD3 was shown to facilitate fibrotic events in the organ beds, signifying the central role this enzyme plays in tissue repair processes. Despite these findings, the role of JMJD3 specifically in wound Mφs and in diabetic wound repair was undefined. Furthermore, the upstream regulation of JMJD3, either in keratinocytes or other cells, in tissue repair has not been explored. We identified a key role for JMJD3 in Mφs in regulating early inflammatory gene expression in normal wounds and that pathologic expression of JMJD3 in late wound Mφs, as seen in diabetes, results in increased late inflammatory gene expression. In both normal and diabetic Mφs, *Jmjd3* expression was regulated by JAK1/3/STAT3, although the ligands regulating this signaling pathway differed. In normal wounds, our group found that IFN-β is elevated at early time points, and this does not occur in diabetic wounds [11]. In this work, we found that this early IFN-β drives *Jmjd3* expression in normal wound Mφs, but in diabetic wounds that have decreased IFN-β after injury, IL-6 acts to drive the JAK1,3/STAT3 signaling pathway.

STING is an endoplasmic reticulum protein that promotes persistent inflammation in malignancy and infection [53, 54]. A recent report detailed the role that STING plays in potentiating lipotoxicity-induced inflammation within pancreatic beta-cells and therefore facilitating the insulin resistance characteristic of diabetes in a murine model [55]. While this and other studies provide a direct link between STING, inflammation, and diabetes/obesity [34, 35], there remains limited information on the role of STING in promoting the chronic inflammatory state observed in diabetic wound repair. Furthermore, STING has not been examined in normal or pathologic wound repair. In particular, wounds, especially nonhealing diabetic wounds, are often a milieu of various pathogens in addition to cellular debris [5] that can serve as ligands for the STING inflammatory pathway. Our study attempts to fill this gap by examining STING in Mφs in wound repair.

STING signaling can proceed by activation of TANK-binding kinase 1 (TBK1) and interferon regulatory factor 3 (IRF3), resulting in the transcription of genes encoding type 1 interferons (IFN-I). [56] The TBK1/IRF3 signaling pathway often counteracts the NFκB pathway, which is responsible for increased proinflammatory cytokine production, such as that of IL-6 and TNF-α. [57, 58] By modulating TBK1/IRF3/IFN-I signaling, STING can act in a dichotomous manner to promote or inhibit inflammation depending on the disease context—a phenomenon that has been previously reported in oncology where chronic STING activation is linked to inflammation-induced carcinogenesis and metastasis via noncanonical NFκB signaling. [59–61]

Our studies indicate that in nondiabetic wound Mφs, *Ifnb* is elevated by Day 1 and gradually diminishes over the wound healing course. This pattern correlates with *Tmem173* expression kinetics in nondiabetic wounds and the increased TBK1/IRF3 signaling observed. Together, these findings confirm the importance of IFN-I in healthy wound healing, as previously reported [11, 62], and suggest that STING is partially responsible for the early increase in IFN-I in nondiabetic wounds, is important for suppressing late inflammatory cytokine production and promotes tissue repair in nondiabetic wounds. However, late and sustained expression of STING in diabetes does not lead to increased STING activation and its downstream IFN-I cytokines. Rather, sustained STING expression in diabetes is detrimental to wound healing due to decreased Mφ-mediated IFN-I production and increased IL-1β, TNF-α, and IL-6 production, all of which act in concert to sustain a proinflammatory Mφ phenotype. We further show that STING is modulated by JMJD3 in diabetic wound Mφs and that blockade of STING in the diabetic setting can lead to decreased inflammation, an increased reparative Mφ phenotype, and improved diabetic wound repair.

Although this study provides valuable insight into the mechanisms behind dysregulated Mφ inflammation in diabetic wounds, some limitations must be addressed. First, within our myeloid-specific JMJD3 murine model, we used the *Lyz2Cre* system. We acknowledge that there is no Cre-specific transgenic line perfect for Mφs given that there is overlap between monocytes/Mφs, neutrophils, and dendritic cells due to their close lineage relationship. Second, we recognize that other epigenetic enzymes may regulate Mφ function in diabetic wounds and that these enzymes do not work in isolation but rather contribute at different phases of wound repair to the cell phenotype [63–65]. Third, we realize that IL-6 is unlikely to be the only driver of JAK1,3/STAT3 signaling in wounds, although IL-6 elevation in diabetic wounds is well documented [66, 67], and we show that it is persistently elevated in diabetic wound Mφs. Finally, we have not directly examined the interplay between Mφs and other structural cells, such as smooth muscle cells or fibroblasts, that exist within the wound. These local interactions clearly impact Mφ signaling and phenotype and likely influence the overall epigenetic program of these cells in wound repair. We acknowledge that studies examining these interactions are necessary to fully understand the factors responsible for impaired diabetic wound healing. We also acknowledge that JMJD3-mediated modulation of Mφ inflammatory activity may affect other cell types involved in the wound healing cascade and that this requires further exploration.

To conclude, our study provides important mechanistic information that JMJD3 regulates inflammation in wound Mφs by decreasing H3K27me3 on inflammatory gene promoters via a JAK1/JAK3/STAT3 pathway. Targeting JMJD3 in a local, Mφ-specific manner is a viable therapeutic strategy that decreases STING-mediated inflammation and other NFκB inflammatory cytokines and improves diabetic tissue repair.

## Materials and Methods

### Mice

Mice were maintained in the University of Michigan Biomedical Sciences and Research Building in the Unit for Laboratory and Animal Medicine (ULAM), which is a pathogen-free animal facility. Mouse experiments were conducted with approval from our Institutional Animal Care and Use Committee (IACUC), and all regulatory and safety standards were strictly adhered to. Male C57BL/6 mice (RRID: IMSR_JAX:000664) were delivered at 6-7 weeks of age from the Jackson Laboratory (Bar Harbor, ME) and were maintained in breeding pairs in the ULAM facilities and were fed a normal chow diet (13.5% kcal fat; LabDiet). Jmjd3^floxed^ mice were created as previously reported by our laboratory. [12] Jmjd3^floxed^ mice were then bred with B6.129P2-Lyz2^tm1(Cre)Ifo/J (Lyz2Cre)^ mice from the Jackson Laboratory to obtain mice deficient in Jmjd3 monocytes, Mφs and granulocytes [68]. Floxed-cre mice were genotyped regularly after birth with custom primers. STING^−/−^ mice were obtained from the Jackson Laboratory (MPYS^-/-^; RRID IMSR_JAX:025805; B6(Cg)-*Sting1*^*tm1.2Camb*^*I*J).

For induction of a diabetic phenotype, male C57BL/6 mice were maintained on a standard high-fat diet (60% kcal saturated fat, 20% proteins, 20% carbohydrate; Research Diets, Inc.) for 12-18 weeks to yield the diet-induced obesity (DIO) model of type 2 diabetes mellitus [69, 70]. After the appropriate time period, HFD-fed (DIO) mice developed obesity and insulin resistance with fasting blood sugars in the mid-200s and elevated insulin levels. All DIO/control animals underwent procedures at 20-32 weeks of age with IACUC approval. For these experiments, only male mice were used, as female mice do not develop DIO.

#### Human wound isolation

All experiments using human samples were approved by the IRB at the University of Michigan (IRB #: HUM00098915) and were conducted in accordance with the principles in the Declaration of Helsinki. Biopsies from human diabetic wounds (*n* = 4) versus normal skin samples (*n* = 38) were collected. The diabetic samples for scRNA-seq were from patients with an average age of 60 years, who all had diabetes (A1c > 7), hypertension, hyperlipidemia, and coronary artery disease. In the nondiabetic patient group, the average age was 70 years, with half the patients having hypertension, hyperlipidemia, and coronary artery disease. Wounds were obtained from the specimens using an 8 mm punch biopsy tool and processed for reverse transcription PCR (RT‒PCR) as described for the murine wounds. RNA with RNA integrity number scores greater than 8 were used, and all values were compared to 28 S/18 S ratios and other housekeeping genes.

### Wound healing

Mice were anesthetized, dorsal hair was removed with Veet (Reckitt Benckiser) and rinsed with sterile water, and two full-thickness back wounds were created by 6-mm punch biopsy with or without wound splinting. The initial wound surface area was recorded, and digital photographs were obtained daily using an 8 mp iPad camera as previously described [71]. Photographs contained an internal scale to allow for standard calibration. The wound area was quantified using ImageJ software (National Institute of Health, Bethesda, MD) and calculated as a percentage of initial wound area. In all pharmacologic dosing experiments, local injection with the drug or vehicle control was performed at four points along the wound edge [20].

### Wound cell digestion

Wounds were harvested from the dorsum of mice postmortem following CO_2_ asphyxiation. Wounds were then minced finely with sharp scissors, and suspensions were placed in a Liberase^TM^ (50 mg/mL; Sigma-Aldrich, St Louis, MO, cat. no. 5101020001) and DNase I (20 U/mL; Sigma-Aldrich, St. Louis, MO, cat. no. 9003-98-9) solution at 37 °C for 30 min for enzymatic digestion. RPMI + FBS was then added to stop the reaction, and the wound cells were then gently plunged and filtered through a 100 μM filter to result in a single cell suspension. Cells were then magnetically sorted for CD3^-^, CD19^-^, NK1.1^-^, Ly6G^-^ and CD11b^+^ cells and cultured ex vivo for RNA, cDNA, or protein studies.

### Magnetic-activated cell sorting (MAC) of murine wounds

MAC sorting of wound cell isolates was performed [72]. Briefly, wound cell isolates were incubated with fluorescein isothiocyanate (FITC)-labeled anti-mouse anti-CD3 (RRID: AB_312660), anti-CD19, and anti-Ly6G (BioLegend) followed by Anti-FITC MicroBeads (Miltenyi Biotec). The resulting flowthrough was then incubated with anti-CD11b MicroBeads to isolate the non-neutrophils, nonlymphocytes, and CD11b^+^ cells. Cells were saved in TRIzol (Invitrogen) for quantitative RT‒PCR analyses.

### Immunofluorescence studies

Isolated wound CD11b^+^ Mφs were incubated on 1.5 mm cover slips under various stimuli by relevant cytokines for 1 h at 37 °C. A 4% paraformaldehyde solution in media was made, and the cells were allowed to fix for 30 min at room temperature. The media solution was aspirated and washed 3x with PBS, and the cover slips were blocked in blocking buffer (1x PBS, 5% fetal bovine serum, 0.3% Triton X-100) for 1 h at room temperature. The primary p-STING antibody (phospho-STING (Ser365) (D1C4T), Cell Signaling Technology) was prepared at a 1:400 concentration in Antibody Dilution Buffer (1x PBS, 1% bovine serum albumin, 0.3% Triton-X100) and applied to the fixed cells overnight at 4 °C. The primary antibody solution was aspirated, and the cells were washed 3 times with PBS and treated with FITC-conjugated anti-rabbit IgG (1:200 v/v; Thermo Fisher) secondary antibody diluted in Antibody Dilution Buffer for 2 h at RT in the dark. Cells were washed and mounted onto microscope slides with Prolong Gold Antifade Reagent with DAPI (Cell Signaling). Slides were allowed to incubate in the dark at RT for 24 h prior to visualization by confocal microscopy (Nikon A1 inverted confocal; 10x magnification). FITC immunofluorescence intensity was analyzed by ImageJ (NIH), and statistical significance was obtained using Student’s t test.

### RNA extraction

Total RNA extraction was performed with TRIzol (Invitrogen) using the manufacturer’s directions. RNA was extracted using chloroform, isopropanol and ethanol. Superscript III Reverse Transcriptase (Thermo Fisher Scientific) kits were used to synthesize cDNA from extracted RNA. cDNA primers for *Jmjd3, Ifnb*, and *Tmem173* were purchased from Applied Biosciences. RT‒PCR was conducted with 2x TaqMan Fast PCR mix and run on a 7500 Real-Time PCR system (Applied Biosciences), and data were then reviewed in a relative quantification analysis to the 18 S ribosomal RNA. All samples were assayed in triplicate. Data were then compiled in Excel (Microsoft) and presented using Prism software (GraphPad v9).

### Chromatin immunoprecipitation assay

Chromatin immunoprecipitation assays were performed as described previously [9]. Briefly, following ex vivo studies, wound Mφs (CD3^-^/CD19^-^/NK1.1^-^/Ly6G^-^/CD11b^+^) were crosslinked in 1% formaldehyde for 10 min at RT, and pellets were stored at -80 °C until analysis. Cells were lysed for 10 min on ice in SDS lysis buffer with a protease inhibitor cocktail (Sigma-Aldrich), syringe passaged and sonicated to generate 100-300 base pair fragments. Five percent of the total chromatin volume was used as the input control. The rest of the chromatin was subsequently incubated with antibodies against trimethylated H3K4, trimethylated H3K9, trimethylated H3K27, or rabbit polyclonal IgG (Millipore Sigma) as a nonspecific antibody control overnight at 4 °C. This step was followed by the addition of Protein A Sepharose beads (Thermo Fisher Scientific) for 1 h at 4 °C. The pellet was washed, and bound DNA eluted 2x for 15 min at room temperature with 5 min at 65 °C at the end of the second elution. The combined eluates were reverse cross-linked for 5 h at 65 °C. Samples were stored at –20 °C, followed by proteinase K digestion for 1 h at 45 °C. DNA was purified using phenol/chloroform/isoamyl alcohol solution. The precipitated DNA was analyzed by quantitative real-time PCR on a TaqMan 7500 sequence detection system. The following primers were used to amplify DNA in samples: *Il1b*: (forward) 5ʹ GCAGGAGTGGGTGGGTGAGT 3ʹ and (reverse) 5ʹ CAGTCTGATAATGCCAGGGTGC 3ʹ, *Tnfa*: (forward) 5ʹ TCCTGATTGGCCCCAGATTG 3ʹ and (reverse) 5ʹ TAGTGGCCCTACACCTCTGT 3ʹ, *Il12*: (forward) 5ʹAGTTAATTCGAAAGCGCCAC 3ʹ and (reverse) 5ʹ CTTTCCCAGGACTGTGTCTC 3ʹ, *Il23:* (forward) 5ʹ GGCTCTCCAAAGAGGGAGAT 3ʹ and (reverse) 5ʹ CCACCTCCTTTGGTTCTGAG 3ʹ, *Nos2a*: (forward) 5ʹ CCAACTATTGAGGCCACACAC 3ʹ and (reverse) 5ʹ GCTTCCAATAAAGCATTCACA 3ʹ, *Tmem173*: (forward) 5ʹAGAAGCCTTTGGCTATCTGG 3ʹ and (reverse) 5ʹ GAGAGATTGAGATGAACAGC 3ʹ.

### Western blot

MAC sorted, CD11b^+^ wound Mφ cells were subjected to cell lysis buffer and protease inhibitor cocktail. Protein suspensions were then standardized for protein concentrations using a Bradford protein assay (Bio-Rad). Equal amounts of protein were mixed with loading buffer and subjected to 4–18% Tris-glycine gel electrophoresis under reducing conditions. Proteins were then wet-transferred at 100 V for 1 hour in Tris-glycine transfer buffer (Invitrogen) to nitrocellulose membranes and probed with primary antibodies (all Cell Signaling; p-JAK1 (D7N4Z), JAK1 (6G4), p-JAK3 (D44E3), JAK3 (D7B12), p-STAT3 (D3A7), STAT3 (D3Z2G), STING (D2P2F), IL-1β (3Α6), β−actin (8H10D10), p-NFκB p65 (Ser536; 93H1), NFκB p65 (D14E12), p-TBK1 (D52C2), TBK1 (E8I3G), p-IRF3 (D6O1M), IRF3 (D83B9) and GAPDH (D16H11)) diluted to 1:500 v/v in 5% bovine serum albumin in Tris buffered saline with Tween buffer overnight at 4 °C with agitation. Nitrocellulose membranes were then washed and incubated with anti-rabbit IgG or anti-mouse IgG HRP-conjugated secondary antibody (Cell Signaling, Inc.) for 1 h at RT with shaking and visualized with timed chemiluminescence (Thermo Fisher Scientific). Densitometry was calculated using ImageJ (NIH), and statistical significance was obtained using unpaired Student’s t test.

### Enzyme-linked immunosorbent assay (ELISA)

Wound Mφs were MACS isolated and stimulated in culture for 4 h in RPMI. After stimulation, the cell-free supernatant was collected and analyzed by specific enzyme immunoassay kits for IL10, IFN-β, IL-1β, and TNF-α (all ELISAs from Cayman Chemical) according to the manufacturer’s instructions.

### Nanoparticle synthesis and application

Nanocarriers containing either dextran-only or dextran-conjugated GSK-J1 (Sigma-Aldrich) *Jmjd3* inhibitor were synthesized. Briefly, in a reaction flask, GSK-J1 (0.2 mmol), dicyclohexylcarbodiimide (0.22 mmol), 4-dimethylaminopyridine (0.05 mmol) and 2-azidoethanol (0.2 mmol) were dissolved in 10 mL of dichloromethane. The mixture was reacted at room temperature for 8 h. The solution was filtered, washed with water (3 times), dried with MgSO_4_, and concentrated for purification via a gel chromatography column with dichloromethane and methanol (0% to 10%) to generate a white or light-yellow solid. Dextran-BCN and the above product (with 3 wt% feeding) were dissolved in DMSO and reacted for 12 h, after which they were precipitated into cold ethanol to obtain the white solid product. Drug loading was determined to be 2.5 wt% via UV‒Vis.

For mouse experiments, the dextran or dextran GSK-J1 conjugated solid was reconstituted in phosphate buffered saline (1 mg/kg) and injected subcutaneously at 4 points along the punch biopsy wounds daily. An 8-megapixel iPad camera with an internal scale was used to record wound size daily. Wound closure was then measured as a percentage of the initial wound area. Images were evaluated by 2 independent, blinded observers. Wound area was calculated using ImageJ software (NIH).

### Histology

Whole wounds were excised from mice or humans using a 6–8 mm punch biopsy. Wound sections were fixed in 10% formalin overnight before embedding in paraffin. Sections (5 μm) were stained with Masson’s trichrome for evaluation of re-epithelialization, granulation and collagen deposition. Images were quantified on ImageScope software and ImageJ at 20X magnification. Percent re-epithelialization was calculated by measuring the distance traveled by epithelial tongues on both sides of the wound divided by the total distance [73].

### Bulk RNA sequencing and scRNA-seq analyses

Generation of single-cell suspensions for scRNA-seq was performed in the following manner: Following informed consent from patients and in accordance with University of Michigan IRB Study # HUM00098915, skin was harvested via punch biopsy from diabetic and nondiabetic control wounds. Samples were incubated overnight in 0.4% Dispase (Life Technologies, Thermo Fisher Scientific) in HBSS (Gibco, Thermo Fisher Scientific) at 4 °C. The epidermal and dermal layers were separated. The epidermis was digested in 0.25% Trypsin-EDTA (Gibco, Thermo Fisher Scientific) with 10 units/mL DNase I (Thermo Fisher Scientific) for 1 h at 37 °C and subsequently quenched with FBS (Atlanta Biologicals) and strained through a 100 μM mesh. The dermis was minced, digested in 0.2% Collagenase II (Life Technologies, Thermo Fisher Scientific) and 0.2% Collagenase V (Millipore Sigma) in plain RPMI medium for 1.5 h at 37 °C, and strained through a 100 μM mesh. Epidermal and dermal cells were combined in a 1:1 ratio for scRNA-seq by the University of Michigan Advanced Genomics Core on the 10x Genomics Chromium System. Libraries were sequenced on the Illumina NovaSeq 6000 sequencer. NovaSeq was used as the sequencing platform to generate 151 bp paired-end reads. We conducted adapter trimming and quality control procedures as described previously [74]. The reads were then mapped using STAR [75] to build human GRCh37, and gene expression levels were quantified and normalized by HTSeq [76] and DESeq2 [77]. Negative binomial models in DESeq2 were used to conduct differential expression analysis. To increase the sample size of the control samples, we used skin biopsies obtained from our previous study [78]. For bulk RNA sequencing and scRNA-seq data accession, the numbers include GSE154556 and GSE154557 (Gene Expression Omnibus). For scRNA-seq data, data processing, including quality control, read alignment, and gene quantification, was conducted using 10X Genomics Cell Ranger software. Seurat was then used for normalization, data integration, and clustering analysis [79]. All clustered cells were mapped to corresponding cell types by matching cell cluster gene signatures with putative cell type–specific markers.

### Statistical analysis

GraphPad Prism software (RRID:SCR_002798) version 9.2.0 was used to analyze the data. Data were analyzed for a normal distribution, and significant differences among multiple groups were obtained using Student’s *t* tests. All *p* values less than or equal to 0.05 were considered significant.

## Supplementary information

Supplemental Figure 1

Supplemental Figure 2

Supplemental Figure 3

Supplemental Figure 4

Supplemental Figure 5

Supplemental Figure 6

Supplemental Figure 7

Supplemental Figure 8

Supplemental Figure 9

Supplemental Figure 10
