# Supplementary figures and images for: Macrophage-specific inhibition of the histone demethylase JMJD3 decreases STING and pathologic inflammation in diabetic wound repair
Source: Cell Mol Immunol. 2022 Sep 20;19(11):1251–62. doi: 10.1038/s41423-022-00919-5 (PMC9622909; doi:10.1038/s41423-022-00919-5)

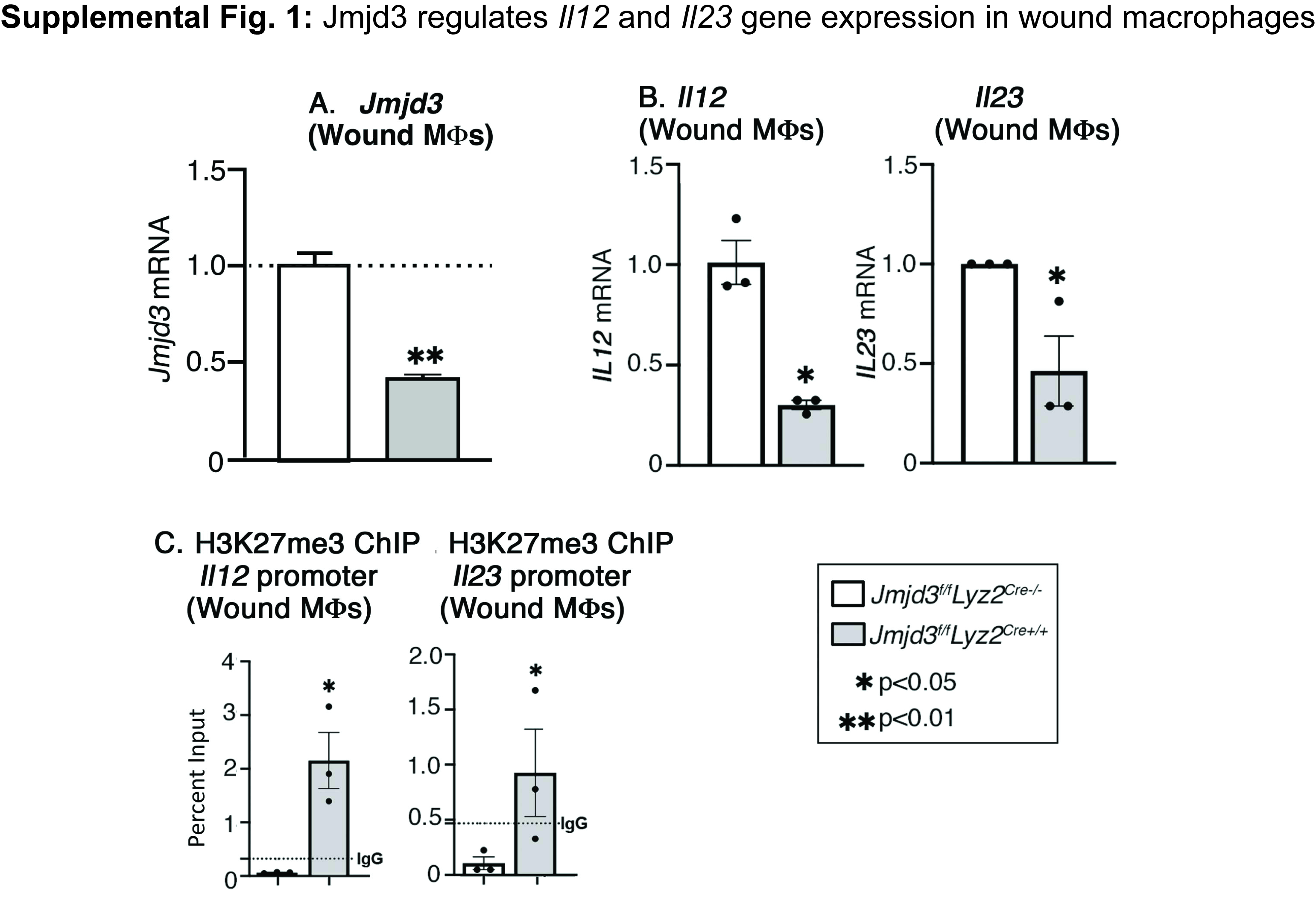

Supplement: Supplementary file 1 — Supplemental Figure 1 [file 41423_2022_919_MOESM1_ESM.tif]

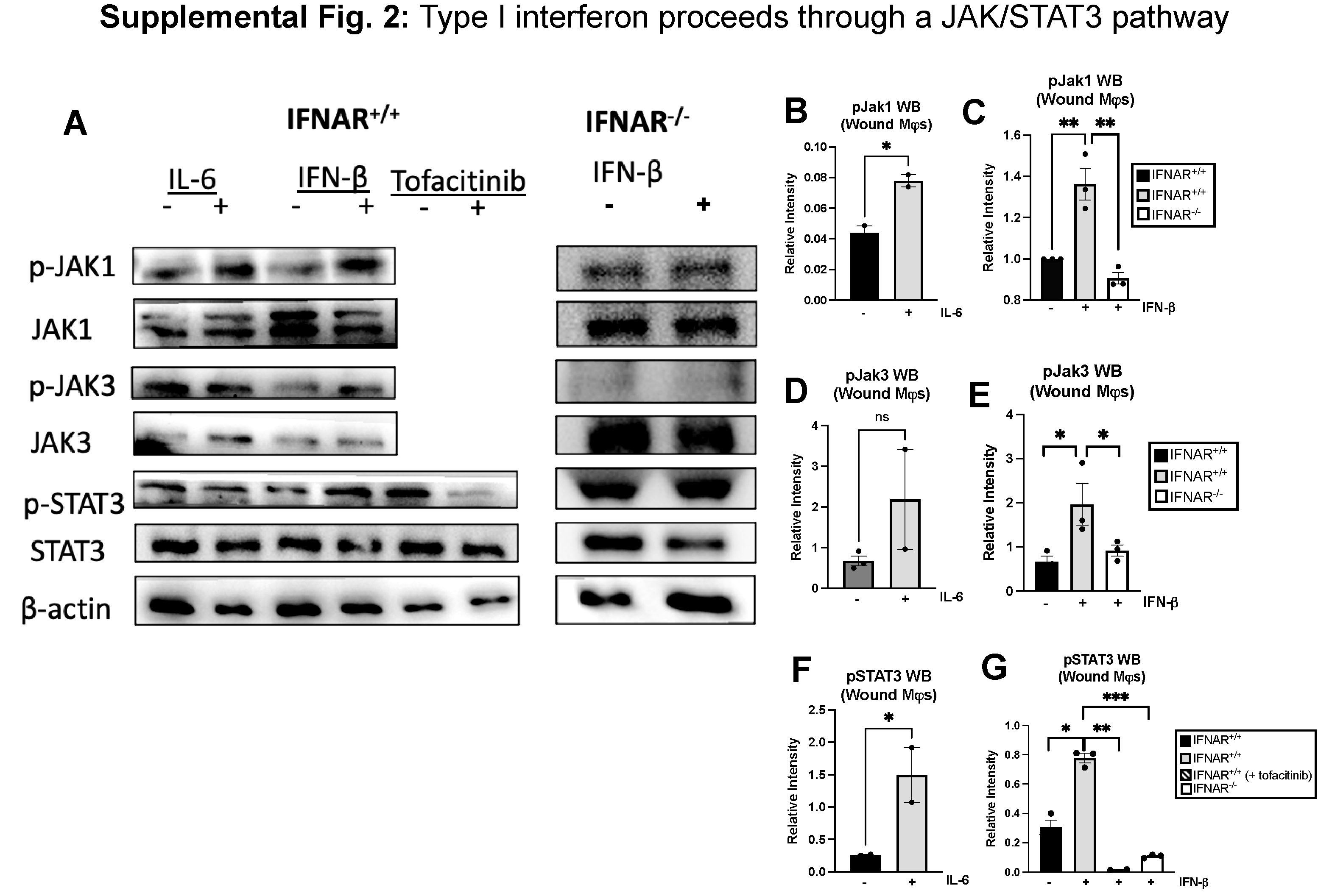

Supplement: Supplementary file 2 — Supplemental Figure 2 [file 41423_2022_919_MOESM2_ESM.tif]

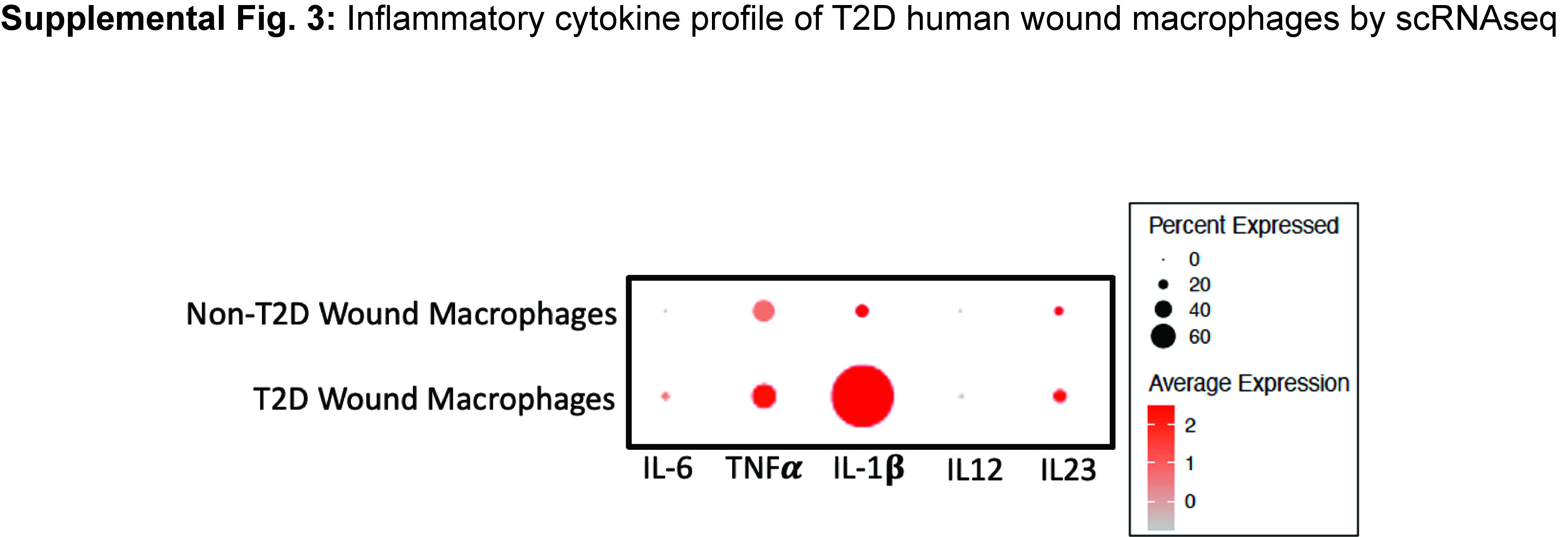

Supplement: Supplementary file 3 — Supplemental Figure 3 [file 41423_2022_919_MOESM3_ESM.tif]

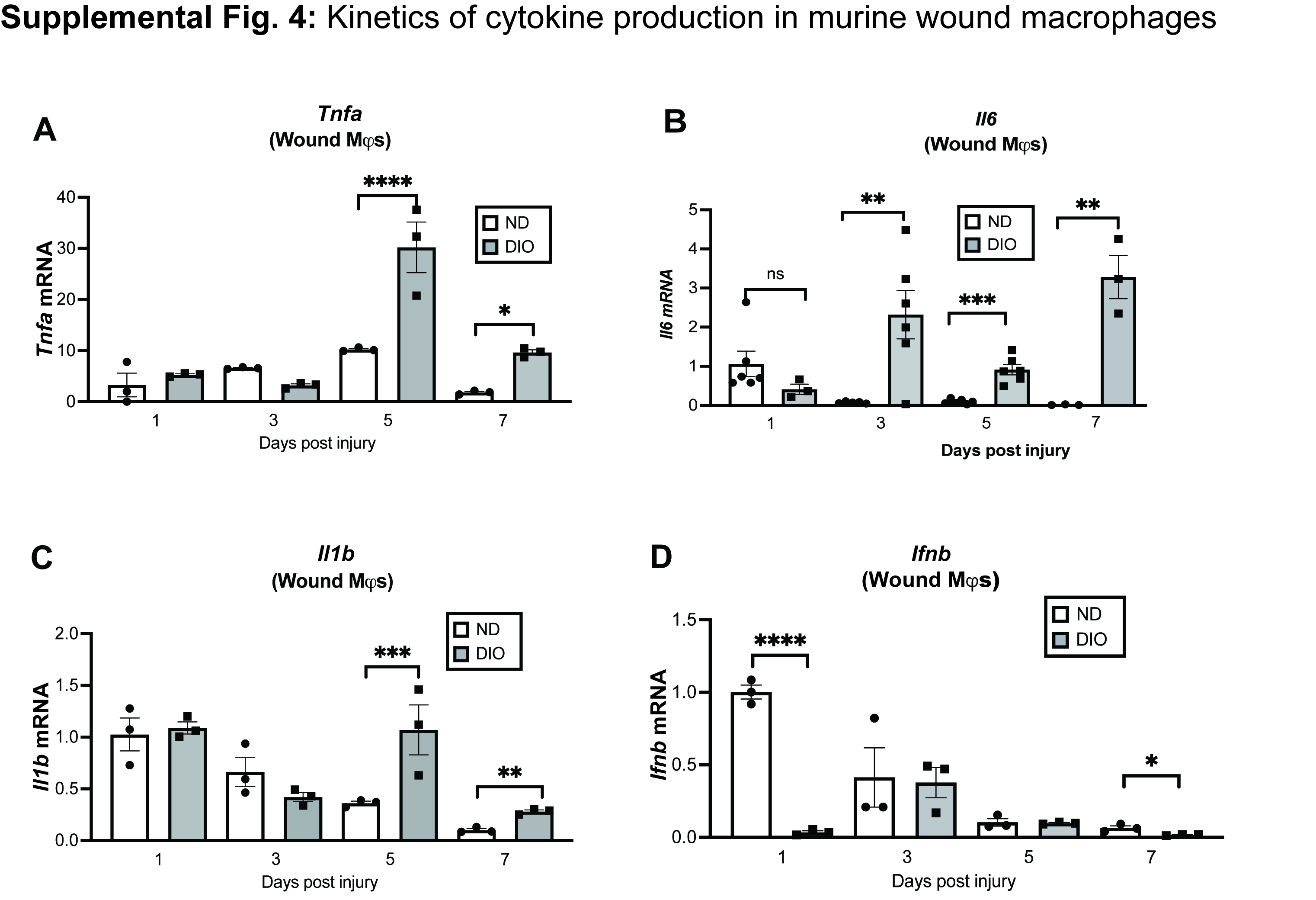

Supplement: Supplementary file 4 — Supplemental Figure 4 [file 41423_2022_919_MOESM4_ESM.tif]

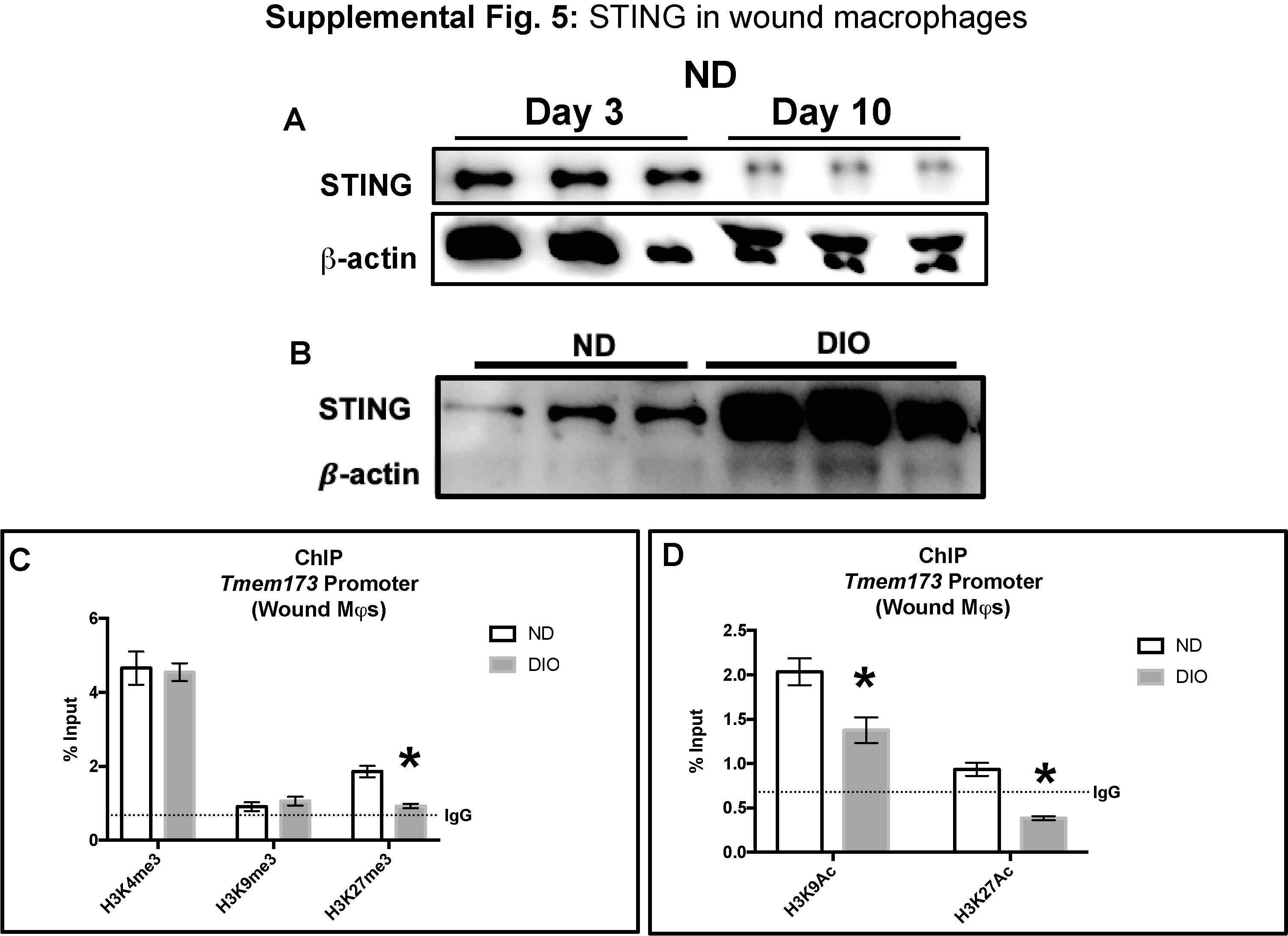

Supplement: Supplementary file 5 — Supplemental Figure 5 [file 41423_2022_919_MOESM5_ESM.tif]

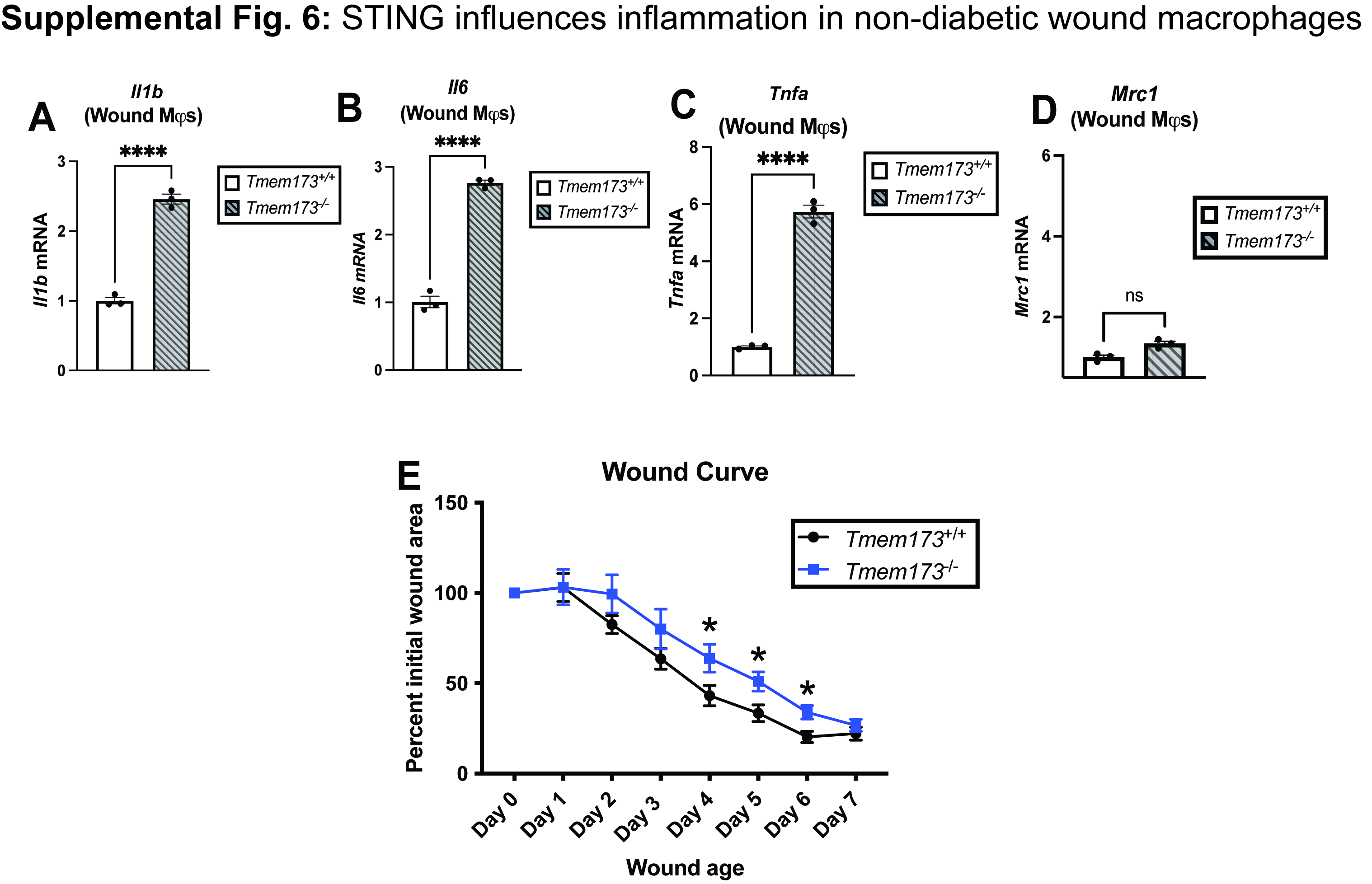

Supplement: Supplementary file 6 — Supplemental Figure 6 [file 41423_2022_919_MOESM6_ESM.tif]

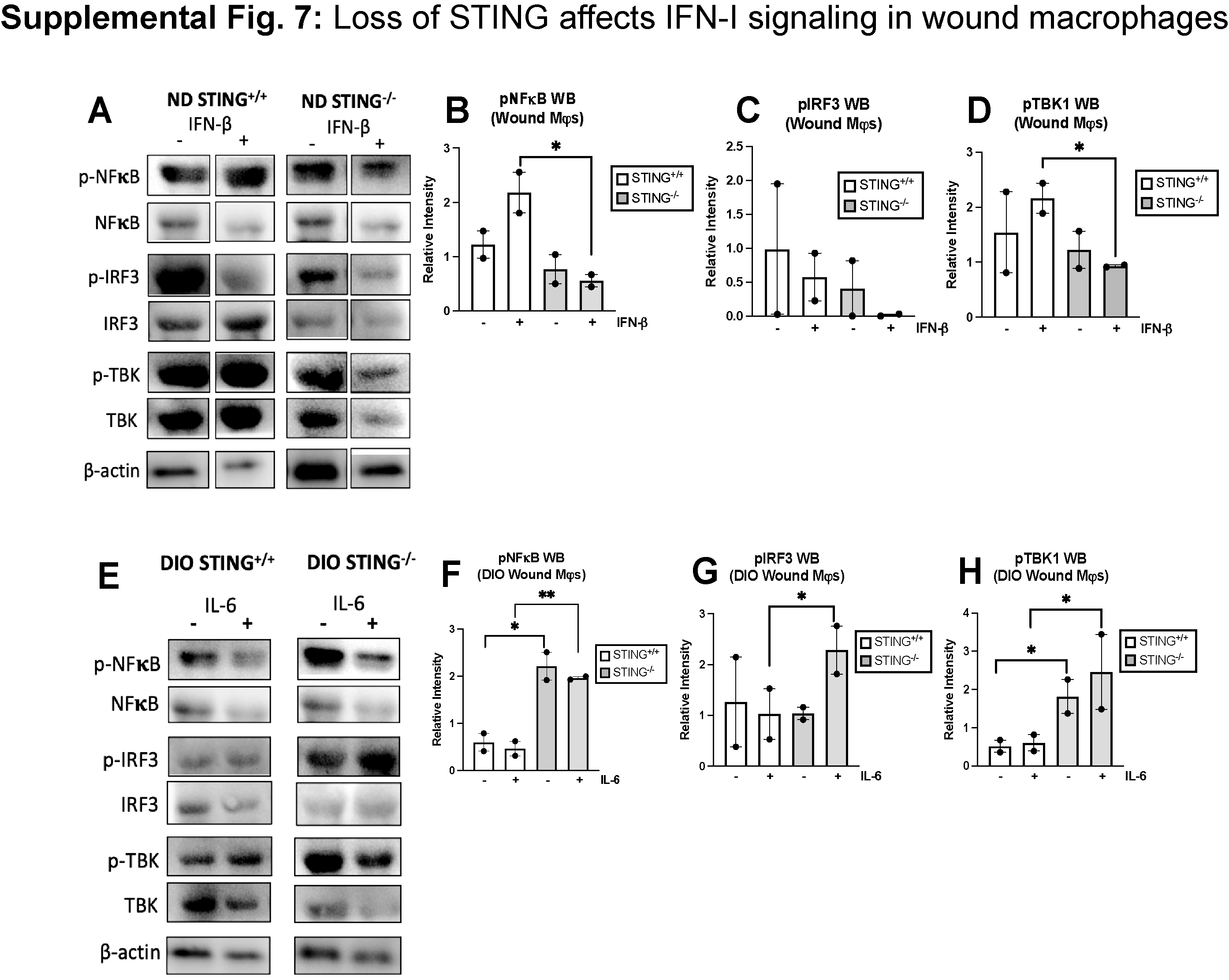

Supplement: Supplementary file 7 — Supplemental Figure 7 [file 41423_2022_919_MOESM7_ESM.tif]

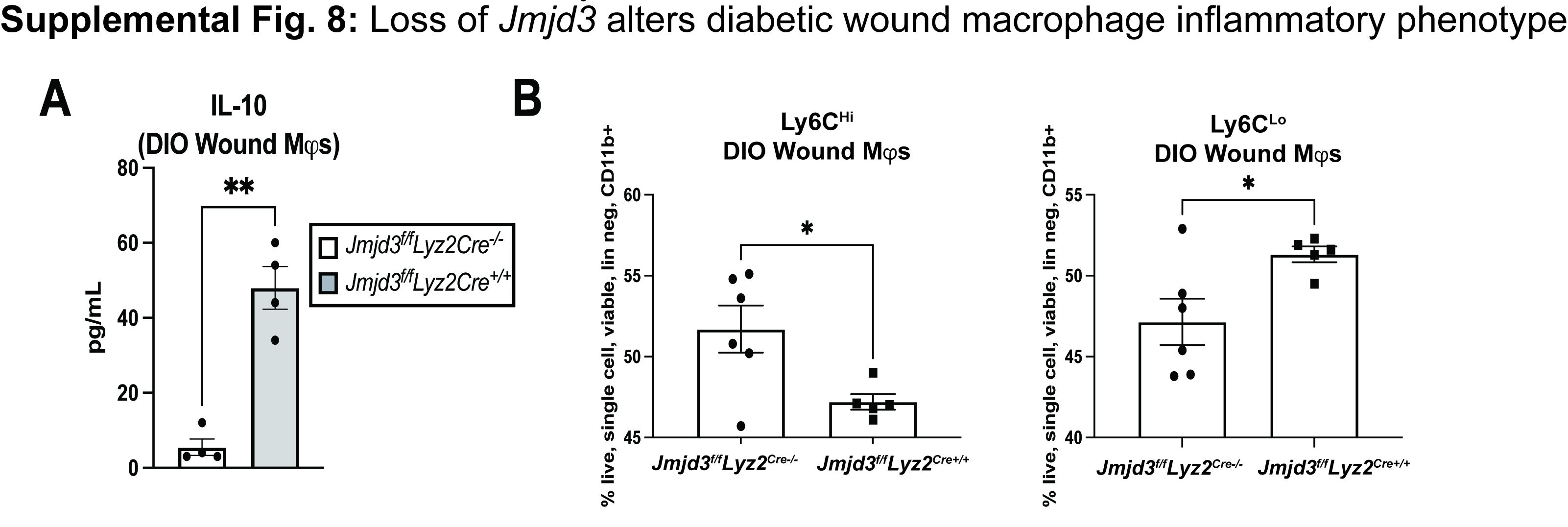

Supplement: Supplementary file 8 — Supplemental Figure 8 [file 41423_2022_919_MOESM8_ESM.tif]

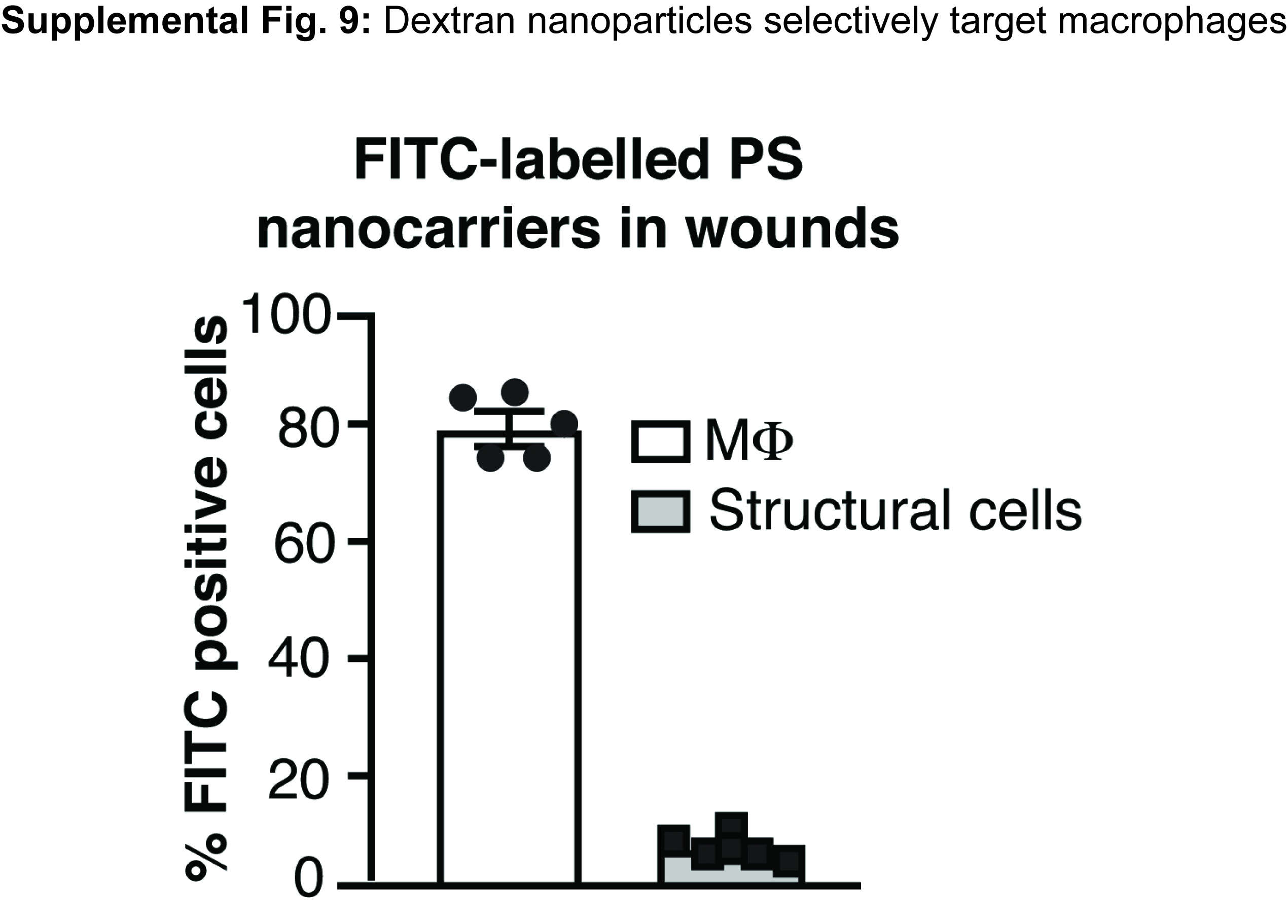

Supplement: Supplementary file 9 — Supplemental Figure 9 [file 41423_2022_919_MOESM9_ESM.tif]

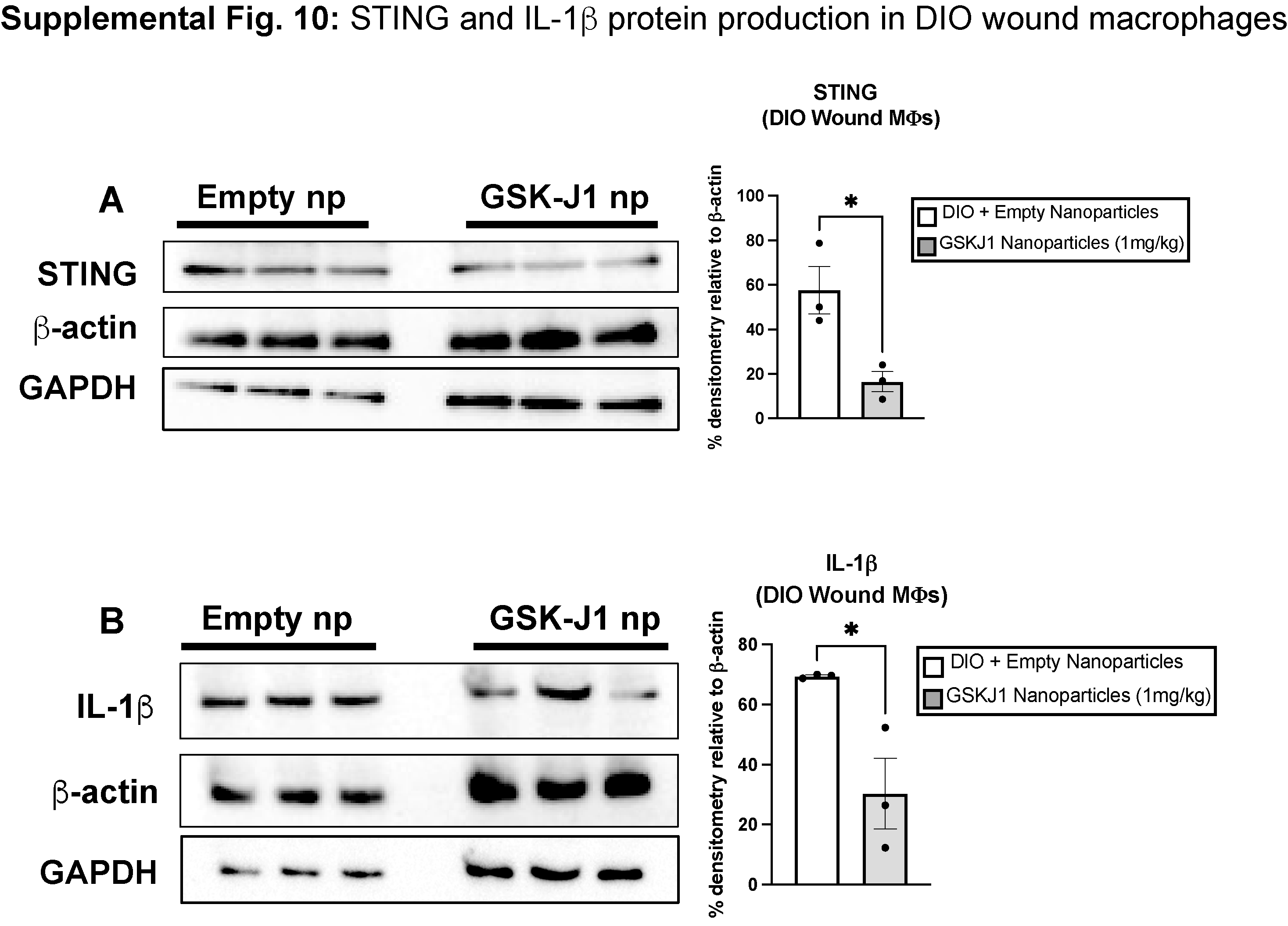

Supplement: Supplementary file 10 — Supplemental Figure 10 [file 41423_2022_919_MOESM10_ESM.tif]
